# Menin Reduces Parvalbumin Expression and is Required for the Anti‐Depressant Function of Ketamine

**DOI:** 10.1002/advs.202305659

**Published:** 2023-12-03

**Authors:** Lige Leng, Kai Zhuang, Hui Lin, Jinjun Ding, Shangchen Yang, Ziqi Yuan, Changquan Huang, Guimiao Chen, Zhenlei Chen, Mengdan Wang, Han Wang, Hao Sun, Huifang Li, He Chang, Zhenyi Chen, Qi Xu, Tifei Yuan, Jie Zhang

**Affiliations:** ^1^ Institute of Neuroscience Department of Anesthesiology The First Affiliated Hospital of Xiamen University School of Medicine Xiamen University Xiamen Fujian 361102 P. R. China; ^2^ Shanghai Mental Health Center Shanghai Jiaotong University School of Medicine Shanghai 200030 P. R. China; ^3^ Department of Geriatrics Xiang'an Hospital of Xiamen university Xiamen Fujian 361102 P. R. China; ^4^ State Key Laboratory of Medical Molecular Biology Institute of Basic Medical Sciences Chinese Academy of Medical Sciences and Peking Union Medical College Neuroscience Center Chinese Academy of Medical Sciences Beijing 100730 P. R. China

**Keywords:** depression, epigenetics, ketamine, menin, parvalbumin

## Abstract

Dysfunction of parvalbumin (PV) neurons is closely involved in depression, however, the detailed mechanism remains unclear. Based on the previous finding that multiple endocrine neoplasia type 1 (Protein: Menin; Gene: *Men1*) mutation (G503D) is associated with a higher risk of depression, a Menin‐G503D mouse model is generated that exhibits heritable depressive‐like phenotypes and increases PV expression in brain. This study generates and screens a serial of neuronal specific *Men1* deletion mice, and found that PV interneuron *Men1* deletion mice (PcKO) exhibit increased cortical PV levels and depressive‐like behaviors. Restoration of Menin, knockdown PV expression or inhibition of PV neuronal activity in PV neurons all can ameliorate the depressive‐like behaviors of PcKO mice. This study next found that ketamine stabilizes Menin by inhibiting protein kinase A (PKA) activity, which mediates the anti‐depressant function of ketamine. These results demonstrate a critical role for Menin in depression, and prove that Menin is key to the antidepressant function of ketamine.

## Graphical abstract

Parvalbumin (PV) increases in several depression mice models. *Men1* deficiency in PV interneurons (PcKO) increases *pvalb* transcription and PV expression by H3K27me3 modification and leading to depression‐like behaviors in mice. The depressive‐like behaviors in PcKO mice can be rescued by PV knockdown or Menin restoring. Ketamine stabilizes Menin protein to contribute its's anti‐depressant function.

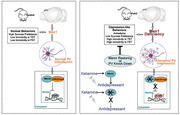

## 1 Introduction

Major depressive disorder (MDD) represents one most prevalent psychiatric illness in the world,^[^ ^1^ ^]^ which associates with significant impairments in mood, social, and occupational functioning, causing substantial health and socioeconomic burdens.^[^ ^2^ ^]^ Despite advances in the understanding of the psychopharmacology and biomarkers of major depression, ≈30% of patients showed limited responses to antidepressant therapy,^[^ ^3^ ^]^ potentially due to the heterogeneity in etiology of MDD. Pathogenic factors of depression involve a combination of environmental and genetic factors.^[^ ^4^ ^]^ Recent genome‐wide association studies (GWASs) have reported ≈100 loci associated with depression.^[^ ^5^ ^]^ Without further animal model verifications, these identified loci may provide useful information regarding to genomic regions for exploring their relevance to depression but have limitations for understanding the depression mechanism.^[^ ^5b^ ^]^

Multiple endocrine neoplasia type 1 (*MEN1*) gene in humans (*Men1* in mice) encodes a scaffold protein: Menin. Loss‐of‐function *MEN1* gene mutations are causal to MEN1 syndrome that is a dominantly inherited disease characterized by tumor formation in endocrine organs.^[^ ^6^ ^]^ Interestingly, MEN1 patients also feature psychiatric symptoms, such as depression.^[^ ^7^ ^]^ We previously identified that carriers of the *MEN1* SNP rs375804228 (a coding mutation conferring G503D) are associated with a higher risk of MDD onset.^[^ ^8^ ^]^ As a nuclear scaffold protein, Menin has diverse functions to positively or negatively regulate gene expression by interacting with number of proteins.^[^ ^6^ ^]^ Among these interaction proteins, histone modifiers account for a large proportion,^[^ ^9^ ^]^ including histone deacetylases 1/2,^[^ ^10^ ^]^ histone H3 lysine 4 (H3K4) methyltransferases,^[^ ^11^ ^]^ histone H3 lysine 27 methyltransferases,^[^ ^12^ ^]^ etc. The methylated H3K27 modification induces chromatin compress, resulting in repression of gene transcription.^[^ ^13^ ^]^

GABAergic interneurons (GABA, γ‐aminobutyric acid) play a major role in neural circuits, cognitive behaviors, and emotion. Among all kinds of interneurons, the proportion of parvalbumin (PV) neurons is the highest, followed by other kinds of interneurons such as neuropeptide somatostatin (SST) and vasoactive intestinal peptide (VIP).^[^ ^14^ ^]^ Multiple lines of evidence have demonstrated that MDD patients or stressed animals exhibit a reduction in cortical GABA levels and in the density of specific GABA interneuron subpopulations, especially PV neurons.^[^ ^15^ ^]^ At the same time, the increased prefrontal GABA transmission in chronic stressed mice was also reported.^[^ ^16^ ^]^ In addition, it has also been reported that chronic unpredictable mild stress (CUMS) and lipopolysaccharide (LPS)‐induced depression mice exhibit increased prefrontal expression of parvalbumin.^[^ ^17^ ^]^ Nevertheless, these opposite observations reflect a high heterogeneity of MDD, and indicate that dysregulated PV expression closely correlates with MDD.^[^ ^14^ ^]^ However, the mechanism underlying the transcription and expression of parvalbumin in depression is largely unclear.

Ketamine is a non‐competitive N‐methyl‐D‐aspartate (NMDA) receptor antagonist that is traditionally used as anesthesia and as antidepressant recently.^[^ ^18^ ^]^ A single dose of ketamine can produce rapid antidepressant response and sustained up to 7 days in patients with refractory depression.^[^ ^19^ ^]^ Tonic‐firing GABA interneurons are more sensitive to low and effective dose of ketamine. Fast‐spiking GABAergic interneurons have faster excitatory postsynaptic potentials (EPSPs) than pyramidal neurons and are more effectively recruited by excitatory inputs. These findings suggest that ketamine initially regulates the spontaneous firing of GABAergic interneurons than pyramidal neurons.^[^ ^20^ ^]^ However, the initial cellular trigger and mechanism of rapid and long‐term antidepressant effects of ketamine remains unclear.

Here, we found that the Menin‐G503D knock‐in mice and conditional PV‐interneuron *Men1* knockout mice (*Men1*‐PcKO) both exhibit depressive‐like behaviors. A significant increase of PV expression was found in the brain of Menin‐G503D mice, *Men1*‐whole‐brain deletion mice, classic CUMS and LPS‐induced depression mice. Menin regulates the PV expression by inhibiting *pvalb* transcription through H3K27me3 modification. The PV knockdown, restoration of Menin expression in PV neurons, or the chemogenetic inhibition of PV cells all attenuated the depressive behaviors in PcKO mice. Furthermore, ketamine can stabilize Menin protein, and fails to attenuate the depressive behaviors in the absence of Menin in *Men1*‐PcKO mice, suggesting that Menin is required for ketamine's antidepressant effects.

## 2 Results

### 2.1 Parvalbumin Expression Increases in the Brain of Menin‐Deficient or G503D Mutant Mice

**Figure 1:**
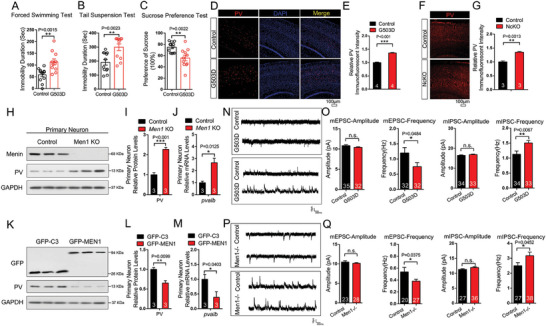
Parvalbumin expression increases in the brain of Menin deficient or G503D mutant mice. A–C) Behavioral analysis of G503D mice and littermate controls using Tail Suspension Tests (TST), Forced Swimming Tests (FST) and Sucrose Preference Tests (SPT). D–G) Immunofluorescence of PV levels in cortex G503D mice (D) and NcKO mice (F), scale bar:100 µm. Quantitation of fluorescence intensity of PV are shown in panel (E and G), *n* = 3‐4 slices from three mice. H–M) The protein and mRNA levels of PV were detected in WT or *Men1* ^−/−^ neurons, and primary neuron transfected with GFP‐C3 or GFP‐*MEN1*. *n* = 3 independent experiments. N–Q) Electrophysiological recording from Menin‐G503D (N,O) and Menin‐NcKO mice (P,Q). Representative whole‐cell recordings on neurons in cortical slices of G503D mice and NcKO mice are shown in panel (N,P), respectively. Quantitation of their mEPSC and mIPSC frequency and amplitude is shown in panel (O,Q), respectively. (n > 20 cells from three mice, respectively). Mouse number used in behavior tests: Control: *n* = 12 mice, G503D: *n* = 12 mice. Data represent mean±SEM, n.s.: not significant, ^*^ *p* < 0.05, ^**^ *p* < 0.01, ^***^ *p* < 0.001. Unpaired *t*‐test for behavioral statistics. Other statistical application between groups were analyzed by one‐way ANOVA with Tukey's post hoc analysis. See also Figures S1,S2,S3 and S4 (Supporting Information).

We previously identified that carriers of the *MEN1* SNP rs375804228 (G503D) are at a higher risk of MDD onset,^[^ ^8^ ^]^ while whether this SNP in *MEN1* alone can cause MDD is still unknown. To test this hypothesis, we created a mouse model harboring the point mutation (G503D) at *Men1* locus by CRISPR/Cas‐mediated genome engineering (S‐**Figure** **1**A). G503D mice were born at an expected mendelian frequency and with a nearly 1:1 sex ratio, and exhibited normal growth rate. The lifespan and their brain morphology and sizes were indistinguishable from controls (S‐Figure 1B–G). No significant difference in the expression of brain Menin was found between G503D with control mice (S‐Figure 1H).

Then, we assessed the potential depressive‐like behaviors in *Men1‐*G503D mice using tail suspension test (TST), forced swimming test (FST), and sucrose preference test (SPT). Notably, G503D mice exhibited depressive‐like phenotypes in all three behavioral tests (Figure 1A–C). We did not observe a significant difference between G503D and control mice in rotarod assay, open field, high‐plus maze, T/Y maze, and Morris water maze tests (S‐Figure 1I–Q). These data indicate that *Men1*‐G503D mice specifically exhibit depressive‐like behaviors.

**Figure 2:**
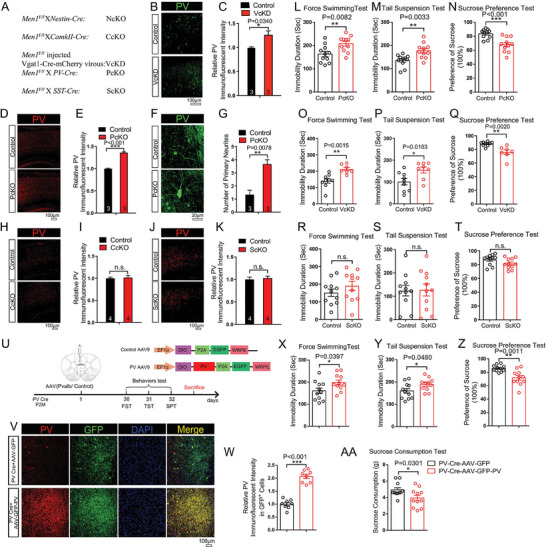
Menin deficiency in interneurons not excitatory neurons increases PV expression and leads to depression‐like behaviors in mice. A) Generation of conditional *Men1* knockout mice by crossing *Men1^F/F^* lines with *Nestin‐cre*, *PV‐Cre, SST‐Cre, or CamKII Cre* mouse lines, refer as: *NcKO, PcKO, ScKO, or CcKO* lines respectively. *Men1^F/F^* mice were injected with *Vgat1 Cre ‐mcherry virus* to get interneuron globally knockdown *Men1* mice (*VcKD*). B,C) Immunofluorescence of PV (green) in cortex from 2‐month‐old *VcKD* mice and controls. Representative confocal images are shown in panel (B), Scale bar:100 µm. Quantitation of immunofluorescent intensity of PV is shown in panel (C), *n* = 3 mice. D–G) Immunofluorescence staining of PV in *PcKO* and control mouse cortex. Representative confocal images are shown in panel (D,F), Scale bar: 100 and 20 µm, respectively. Quantitation of fluorescence intensity and primary neuritis of PV are shown in panel (E,G), *n* = 3 mice. H–K) Immunofluorescence of PV levels in cortex from 2‐month‐old *CcKO* mice (H, I) or *ScKO* mice (J,K). Representative confocal images are shown in panel (H,J), Scale bar:100 µm. Quantitation of fluorescence intensity of PV are shown in panel (I,K), *n* = 4 mice. L–N) Behavioral analysis of *PcKO* and littermate controls using FST, TST, and SPT. O–Q) Behavioral analysis of *VcKD* mice and littermate controls using FST, TST and SPT. R–T) Behavioral analysis of *ScKO* mice and littermate controls using FST, TST, and SPT. U) Schematic diagram of the structure of the PV‐AAV and associated workflow in mice. V,W) Double immunofluorescence staining to detect GFP (green) and PV (red) in PV Cre+AAV‐GFP, and PV Cre+AAV‐GFP‐PV mice cortex. Representative confocal images are shown in panel (V), Scale bar:100 µm. Quantitation of fluorescence intensity of PV in GFP^+^ cells is shown in panel (W), *n* = 3 mice. X–AA) Behavioral analysis of AAV‐GFP‐PV or AAV‐GFP injected PV‐Cre mice by TST, FST, SPT, and SCT. Mouse number used in behavior tests: Control: *n* = 9 mice, *VcKD*: *n* = 8 mice. Control: *n* = 11 mice, *PcKO*: *n* = 11 mice; Control: *n* = 11 mice, *ScKO*: *n* = 11mice. PV Cre+AAV‐GFP: *n* = 11 mice, PV Cre+AAV‐GFP‐PV: *n* = 11 mice. Data represent mean±SEM, ^*^ *p* < 0.05, ^**^ *p* < 0.01, ^***^ *p* < 0.001. Unpaired t‐test for behavioral statistics. Other statistical application between groups were analyzed by one‐way ANOVA with Tukey's post hoc analysis. See also Figures S5,S6,S7,S8 and S9 (Supporting Information).

**Figure 3:**
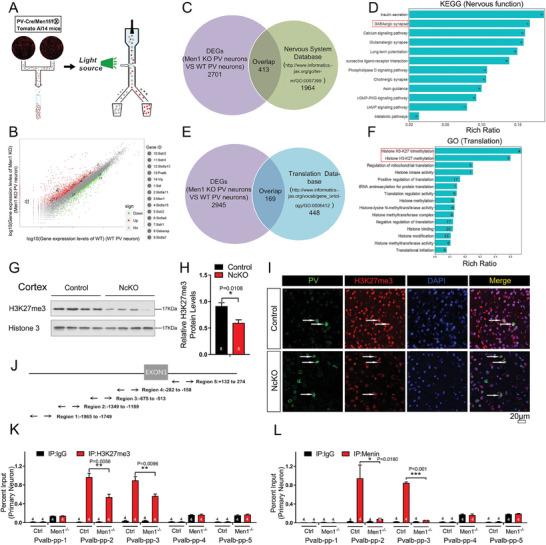
Transcriptome analysis of *Men1* ^−/−^ PV neurons revealed epigenetics regulation of *pvalb* by Menin. A) *PcKO* mice were crossed with Ai14 tomato reporter mice, and fluorescent PV cells from the brain were subjected for flow cytometry sorting. B–F) Transcriptome sequencing of *Men1* knockout and WT PV neurons. Differentially expressed genes (DEGs) were identified and were shown in panel (B). C–F) These DEGs were compared with the nervous system database or the translation database of mouse genome information, and the overlapped genes were subjected to KEGG pathway analysis or GO pathway analysis. G,H) Western blot analysis of H3K27me3 protein expression in primary neuron from *NcKO* and control mice. Quantification of protein levels is shown in (H), n = 8 mice. (I) Double immunofluorescence staining to detect PV (green) and H3K27me3 (red) in *NcKO* and control mice cortex. Scale bar:20 µm, *n* = 3 mice. J) Schematic diagram of *pvalb* promoter region. K,L) ChIP assays using antibodies against H3K27me3 (K) or Menin (L) were performed in cultured *Men1*‐knockout or WT neurons on DIV 12. *n* = 4 independent experiments.Data represent mean±SEM. ns: not significant, ^*^ *p* < 0.05, ^**^ *p* < 0.01, ^***^ *p* < 0.001, one‐way ANOVA with Tukey's post hoc analysis. See also Figure S10 (Supporting Information).

**Figure 4:**
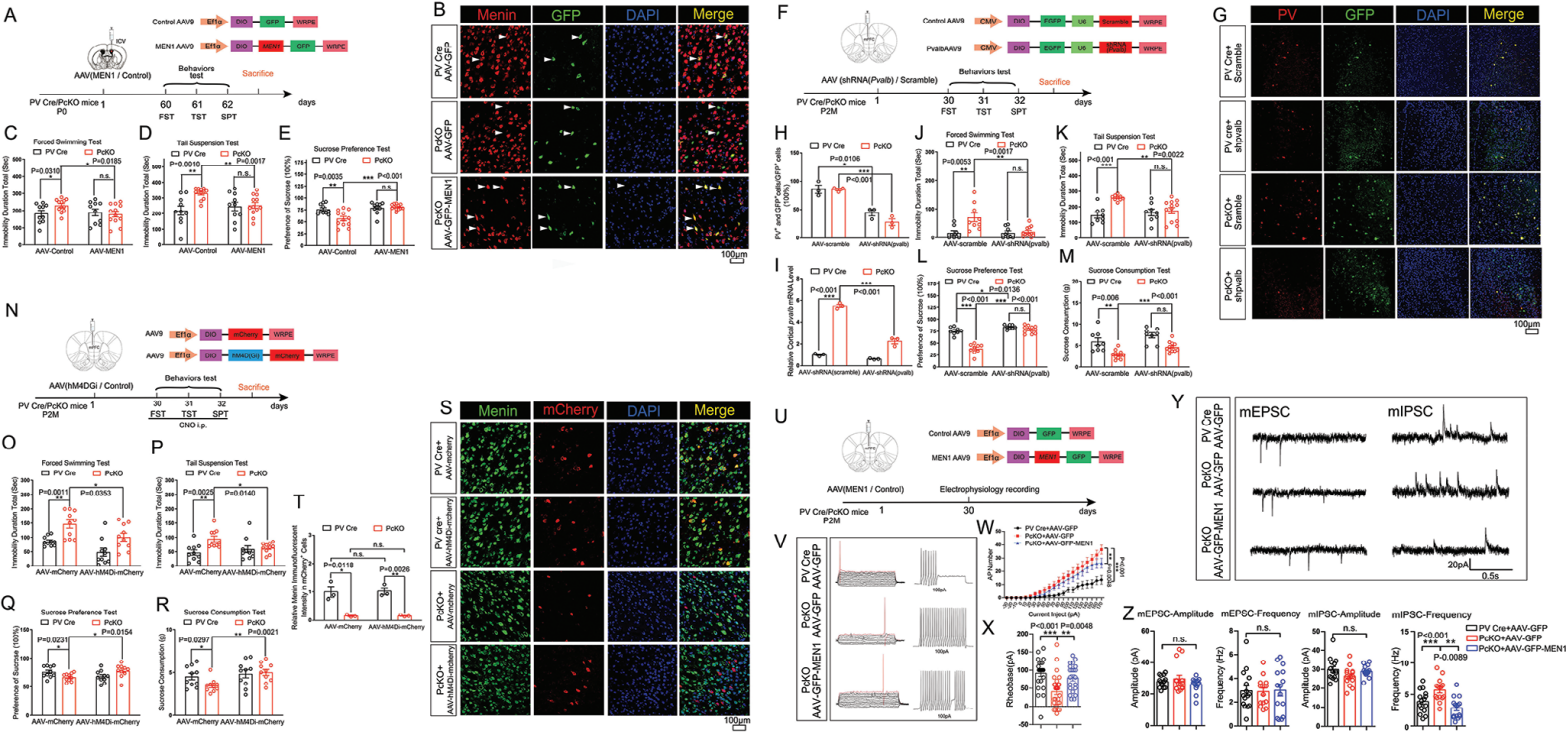
Menin restoration, PV downregulation or PV neuronal activity inhibition in PV interneurons all rescue depressive behaviors in *Men1*‐PcKO mice. A) Schematic diagram of the structure of the *MEN1*‐AAV and associated workflow in mice. B) Double immunofluorescence staining to detect GFP (green) and Menin (red) in PV Cre+AAV‐GFP, PcKO+AAV‐GFP and PcKO+AAV‐GFP‐*MEN1* mice cortex. Representative confocal images are shown in panel (B), Scale bar:100 µm. *n* = 3 mice. C–E) Behavioral analysis of AAV‐GFP‐MEN1 or AAV‐GFP injected PcKO and PV‐Cre mice by TST, FST, and SPT. F) Schematic diagram of the structure of the *shpvalb*‐AAV and associated workflow in mice. G,H) Immunofluorescence staining of PV and GFP in mPFC of mice received virous injection as labeled in panel G, Scale bar:100 µm. Quantitation of fluorescence intensity of PV in GFP^+^ cells is shown in panel (H), *n* = 3 mice. I) The mRNA levels of *pvalb* were measured by quantitative RT‐PCR in the cortex of the above mice, *n* = 3 mice. J–M) Behavioral analysis of AAV‐EGFP‐shRNA*(Pvalb)* or AAV‐EGFP‐shRNA*(scramble)* treated PcKO and littermate controls by FST, TST, SPT, and Sucrose Consumption Tests (SCT). N) Schematic diagram of the structure of the AAV‐hSyn‐DIO‐hM4D(Gi) and AAV‐hSyn‐DIO and associated workflow in mice. O–R) Behavioral analysis of AAV‐hSyn‐DIO‐hM4D(Gi) and AAV‐hSyn‐DIO injected *PcKO* and PV‐Cre mice by TST, FST, SPT and SCT. S,T) Immunofluorescence staining of Menin and mCherry in mPFC of mice received virous injection as labeled in panel (S), Scale bar:100 µm. Quantitation of fluorescence intensity of Menin in mCherry^+^ cells is shown in panel (T), *n* = 3 mice. U) Schematic diagram of the structure of the *MEN1*‐AAV and associated workflow in mice. V–X) Electrophysiological current‐clamp traces of action potential of PV interneurons from *PV cre*+AAV‐GFP, *PcKO*+AAV‐GFP and *PcKO*+AAV‐GFP‐*MEN1* mice brain slices are shown in panel (V). Input‐output plot of PV interneurons from *PV cre*+AAV‐GFP, *PcKO*+AAV‐GFP and *PcKO*+AAV‐GFP‐*MEN1* mice are shown in panel (W). The analysis of PV interneuron rheobase are shown in panel X (n>20 neurons from 3–6 mice each group). (Y, Z) Electrophysiological recording of mEPSC and mIPSC of PV interneuron from PV Cre+AAV‐GFP, PcKO+AAV‐GFP and PcKO+AAV‐GFP‐*MEN1* mice. Representative whole‐cell recordings on PV interneurons in cortical slices of PV Cre+AAV‐GFP, PcKO+AAV‐GFP and PcKO+AAV‐GFP‐*MEN1* mice are shown in panel (Y). Quantitation of their mEPSC and mIPSC frequency and amplitude are shown in panel (Z). (*n* = 15 cells from three mice each group) Mouse number used in behavior tests: *PV cre*+AAV‐GFP: *n* = 10 mice, *PcKO*+AAV‐GFP: *n* = 12 mice, *PV cre* + AAV‐GFP‐*MEN1*: *n* = 10 mice, *PcKO*+AAV‐GFP‐*MEN1*: *n* = 12 mice. *PV cre*+AAV‐EGFP‐shRNA(Scramble): *n* = 8 mice, *PcKO*+AAV‐EGFP‐shRNA(Scramble): *n* = 11 mice, *PV cre* + AAV‐EGFP‐shRNA(*pvalb*): *n* = 8 mice, *PcKO*+AAV‐ EGFP‐shRNA*(pvalb*): *n* = 13 mice. *PV cre*+ AAV‐hSyn‐DIO: *n* = 10 mice, *PcKO*+ AAV‐hSyn‐DIO: *n* = 10 mice, *PV cre* + AAV‐hSyn‐DIO‐hM4D(Gi): *n* = 10 mice, *PcKO*+ AAV‐hSyn‐DIO‐hM4D(Gi): *n* = 10 mice. Data represent mean±SEM, ^*^ *p* < 0.05, ^**^ *p* < 0.01, ^***^ *p* < 0.001. Unpaired t‐test for behavioral and electrophysiological statistics. Other statistical application between groups were analyzed by one‐way ANOVA with Tukey's post hoc analysis. See also Figure S11 (Supporting Information).

The RNA sequencing analysis of cortex of G503D and control mice revealed a total of 414 differentially expressed genes (DEGs) (S‐**Figure** **2**A; Dataset S1, Supporting Information). The DEGs were compared with the mouse genome CNS database, and 111 DEGs related with CNS were selected. Gene ontology (GO) biological process analysis of these genes showed that interneuron‐related, especially PV‐related modulation pathways were significantly enriched (S‐Figure 2B,C). These results prompted us to assess the density of inhibitory interneurons in *Men1*‐G503D brains. We observed significantly increased PV expression in Menin‐G503D cortices compared with controls at P60 by immunostaining and RT‐PCR (Figure 1D,E; S‐Figure S1R,S, Supporting Information). Other types of interneurons including calbindin^+^, SST^+^, and VIP^+^ were found no difference in G503D cortices compared with controls (S‐**Figure** **3**A–F). Meanwhile, the density of NeuN^+^ neurons and GFAP^+^ astrocytes were also observed no difference between G503D and controls’ cortices (S‐Figure 3G–J). Increased immunofluorescent intensity of Iba1 (S‐Figure 3K,L) and enhanced neuroinflammation factors (S‐Figure S1T,U, Supporting Information) were found in G503D cortices, which is consistent with our previous finding that Menin‐G503D mutation may activate neuroinflammation by NF‐κB.^[^ ^8^ ^]^ In addition, we also observed increased PV levels in CUMS‐treated mice (S‐**Figure** **4**).

**Figure 5:**
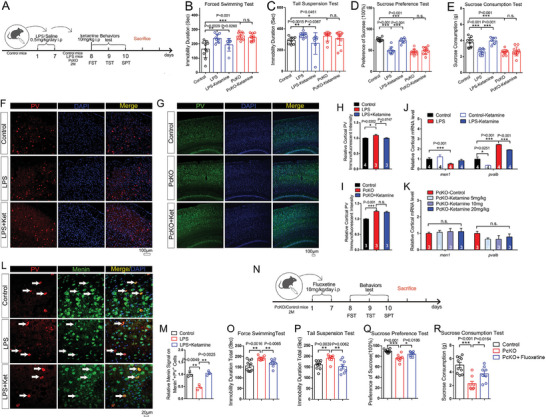
The antidepressant effect of ketamine requires Menin. A) Schematic diagram of ketamine administration (intraperitoneal administration) and associated workflow in LPS model mice, *PcKO* and littermate control mice. B–E) Behavioral analysis of ketamine or saline‐treated LPS model mice, *PcKO* mice and littermate controls by TST, FST, SPT, and SCT. F–I) Immunofluorescence of PV in cortex from LPS model mice, *PcKO* mice and littermate controls. Representative confocal images are shown in panel (F, G), Scale bar:100 µm. Quantitation of fluorescence intensity of PV is shown in panel (H,I), *n* = 3‐4 slices from three mice. J,K) Quantitative RT‐PCR measurements to determine *men1* and *pvalb* levels in cortex of the above mice (J) and different dose of ketamine treated PcKO mice (K), *n* = 3–4 mice. L,M) Immunofluorescence of PV (red) and Menin (green) in cortex of control mice, LPS model mice and LPS+ketamine mice. Representative confocal images are shown in panel (L), Scale bar: 20 µm. Quantitation of relative fluorescence intensity of Menin on Menin^+^ + PV^+^ cells is shown in panel (M), *n* = 3 mice. N) Schematic diagram of fluoxetine administration (intraperitoneal administration) and associated workflow in *PcKO* and littermate control mice. O–R) Behavioral analysis of fluoxetine or saline‐treated *PcKO* mice and littermate controls by TST, FST, SPT, and SCT. Mouse number used in behavior tests: Control: *n* = 10 mice, LPS mice: *n* = 9 mice, LPS mice+ketamine: *n* = 7 mice, *PcKO*: *n* = 10 mice, *PcKO*+ketamine: *n* = 11 mice. Control: *n* = 10 mice, *PcKO*: *n* = 8 mice, *PcKO*+fluoxetine: *n* = 8 mice. Data represent mean±SEM, ^*^ *p* < 0.05, ^**^ *p* < 0.01, ^***^ *p* < 0.001. Unpaired *t*‐test for behavioral statistics. Other statistical application between groups were analyzed by one‐way ANOVA with Tukey's post hoc analysis.

In consistent with the findings in Menin‐G503D mice, the elevated PV staining activity (Figure 1F,G) and depression‐like behaviors^[^ ^8^ ^]^ were also observed in Menin brain‐specific knockout mice (*NcKO*). And no changes were found in the densities of calbindin^+^, somatostatin^+^, and VIP^+^ cells in NcKO brains compared with controls (S‐**Figure** **5**A–F). We then measured the PV expression in *Men1*‐KO neurons and found that the mRNA and protein levels were both increased in the absence of Menin (Figure 1H–J). Vice versa, the PV expression was decreased in primary neurons with *MEN1* overexpression (Figure 1K–M). We further examined whether the GABAergic synaptic activity is altered in these mice. Electrophysiology recording was performed in neurons from Menin‐G503D and NcKO (*Men1* ^−/−^) (P20) brain slices. No changes were found in amplitude of miniature excitatory postsynaptic currents (mEPSC) between G503D neurons, *Men1* ^−/−^ neurons with their controls, however, the frequency of mEPSC is decreased in both G503D neurons and *Men1* ^−/−^ neurons compared with controls, respectively. In contrast, the frequency of miniature inhibitory postsynaptic currents (mIPSC) was increased in both G503D neurons and *Men1* ^−/−^ neurons, compared with their controls (Figure 1N–Q). These results indicate that the Menin mutation or deficiency‐induced PV expression leads to the dysfunction of interneurons, which may contribute to the pathogenesis of depression.^[^ ^15a^ ^]^

### 2.2 Menin Deficiency in Interneurons Especial in PV Neurons Leads to Increased PV Expression and Depressive Behaviors in Mice

**Figure 6:**
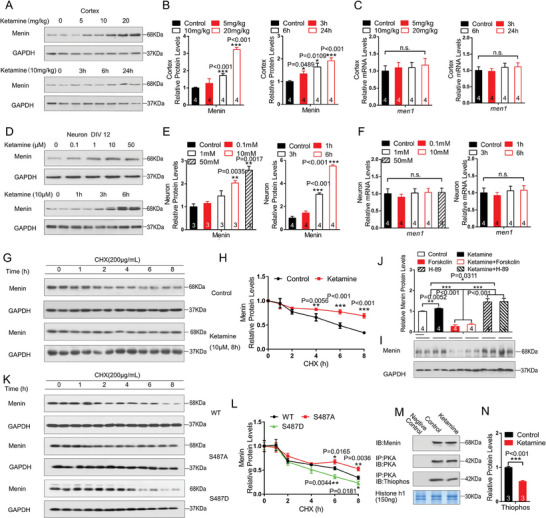
Ketamine stabilizes Menin through inhibiting PKA‐mediated phosphorylation. A–C) WT mice were exposed to ketamine for 60 min at various concentrations (A) or for various times at 10 mg kg^−1^. The protein and mRNA levels of Menin were measured by western blotting or RT‐PCR, *n* = 4 mice. D–F) Primary cultured neurons were treated with ketamine for 60 min at various concentrations or for various times at 10 µM. The protein and mRNA levels of Menin were measured by western blotting or RT‐PCR, *n* = 3–4 independent experiments. G,H) HEK293T cells were pre‐treated with ketamine or vehicle control, and then were exposed to cycloheximide for the time indicated. The Menin degradation rates were determined by western blotting. Representative blots are shown in panel (G). Quantification of Menin protein turnover is shown in panel (H), *n* = 3 independent experiments. I,J) Primary neurons were exposed to ketamine, PKA agonist forskolin or PKA antagonist H89, the protein levels of Menin were detected by western blotting, the representative blots are shown in panel (I) and their quantifications were shown in panel (J), *n* = 4 independent experiments. K,L) HEK293T cells were transfected with GFP‐Menin (S487A) and GFP‐Menin (S487D). The cells were then exposed to cycloheximide for the time indicated. The Menin degradation was determined by western blotting. Representative blots are shown in panel (K). Quantification of Menin protein turnover is shown in panel (L), *n* = 3 independent experiments. M,N) In vitro PKA kinase activity assay in brain lysates from mice treated with ketamine. The brain lysates were co‐immunoprecipitated by PKA antibody and followed anti‐thiophosphate ester antibody assay with histone‐H1 as substrate. The levels of histone‐H1 were visualized using Coomassie blue staining, *n* = 3 independent experiments. Data represent mean±SEM. ns: not significant, ^*^ *p* < 0.05, ^**^ *p* < 0.01, ^***^ *p* < 0.001, one‐way ANOVA with Tukey's post hoc analysis.

We then investigate the function of Menin in interneurons, particularly in PV neurons. We generated a serial *Men1* conditional knockout mice: *Men1* floxp mice (*Men1* ^F/F^) were crossed with *PV‐Cre*, or *Somatostatin‐Cre* mice to generate *Men1*‐PV neuron deletion mice: PcKO, or *Men1*‐SST neuron deletion mice: ScKO; rAAV‐vGAT1‐Cre‐mCherry‐WPRE‐pA virus was injected into P0 *Men1* ^F/F^ mice to globally knockdown *Men1* in all interneurons,^[^ ^21^ ^]^ which was named: VcKD (vGAT1‐cre mediated Menin knockdown) (Figure 2A). We previously generated *CamKII‐Cre‐Men1* ^F/F^ (CcKO) mice for excitatory neuron *Men1* deletion.^[^ ^8^, ^22^ ^]^ The *Men1‐* VcKD, PcKO and ScKO animals exhibited indistinguishable body and brain sizes with their controls (*Men1^F/F^* animals were used as controls) (S‐**Figure** **6**). The efficiency of Menin knockdown or deletion in VcKD, PcKO, and ScKO mice was confirmed by Menin staining (S‐Figure S7A–C, Supporting Information).

We then assessed the PV expression and the depression‐like phenotypes in these mice. We found that the PV immunostaining intensity increased in both VcKD and PcKO mice cortex compared with controls (Figure 2B–E; S‐Figure S8G–J, Supporting Information). Further, the neurite length and number of primary neurites of PV^+^ cells were also increased in *Men1*‐PcKO cortex (Figure 2F,G). We also found that the PV expressions increased in different type neurons in PcKO mice brain (S‐Figure S8A–F, Supporting Information). However, we found no changes in the density of calbindin^+^, somatostatin^+^, and VIP^+^ cells in *Men1*‐PcKO cortices, compared with controls (S‐Figure 5G–L). In contrast, we didn't observe significant changes in the PV^+^ neuronal density in the cortex of *Men1*‐ScKO and *Men1*‐CcKO compared with their controls (Figure 2H–K). To ascertain the functional relevance of the morphological and biochemical changes in interneuron Menin deficiency mice, *Men1*‐PcKO, VcKD and ScKO mice were subjected for locomotive, cognitive, social and emotional behaviors tests. Notably, both PcKO and VcKD mice exhibited significant depressive like behaviors in TST, FST and SPT tests (Figure 2L–Q). PcKO mice behaved normally in rotarod, elevated plus maze, and Morris water maze tests, while were found to be impaired in the presence of novel interaction partners during social interaction tests (SITs) (S‐Figure S9A–H, Supporting Information). No significant increase of neuroinflammation factors (S‐Figure S9I,J, Supporting Information) were found in PcKO mice cortex and hippocampus compared with the control mice. We did not observe depressive‐like behaviors in mice with Menin deficiency in somatostatin neurons (ScKO) (Figure 2R–T). To confirm whether enhanced PV overexpression in PV interneurons can directly lead to the depressive‐like behaviors, exogenous PV was expressed specifically in PV interneurons by injecting Cre recombinase‐dependent PV‐AAV into the mPFC of *PV cre* mice. Behavioral tests were subsequently performed 30 days after injection (Figure 2U). Notably, PV overexpression in PV cells significantly leads to increased PV expression as measured by immunofluorescence staining (Figure 2V,W), as well as the depressive‐like behaviors in TST, FST, SPT and SCT tests (Figure 2X–AA). These results demonstrate that mice with downregulation of Menin or overexpression PV in PV interneurons manifests depressive‐like phenotypes.

### 2.3 Menin Deficiency Upregulates PV Expression Epigenetically

To investigate the regulating mechanism of Menin on PV expression, the transcriptome analysis of wild‐type and Menin deletion PV neurons were performed. To collect PV neurons, *Men1* PcKO mice were crossed with Tomato Ai14 tracing mice^[^ ^23^ ^]^ to label PV interneurons with mCherry fluorescence. The mCherry^+^ PV cells were then purified by fluorescence‐activated cell sorting (FAC‐sorting) and subjected to RNA‐Seq analysis (Figure 3A; Dataset S2, Supporting Information). A total of 3114 differentially expressed genes (DEGs) between the *Men1* knockout PV cells with control PV cells were identified, the significantly changed GABAergic genes were labeled and listed (Figure 3B). Comparison of these DEGs with the nervous system database of mouse genome information (http://www.informatics.jax.org/go/term/GO0007399) revealed 413 overlapped genes (Figure 3C). The Kyoto Encyclopedia of Genes and Genomes (KEGG) analysis found that the genes downregulated by *Men1* in PV interneurons are highly clustered in GABAergic associated pathway (Figure 3D). Since Menin is a well‐known epigenetic and transcriptional co‐factor,^[^ ^6^, ^9^, ^11^ ^]^ we compared the 3114 DEGs with the mouse translation database (http://www.informatics.jax.org/go/term/GO:0006412) (Figure 3E), and found 169 overlapped genes. GO analysis revealed that these genes are highly enriched in histone modification, specifically in histone H3‐K27 methylation (Figure 3F).

We found that the H3K27me3 level in Menin deficient mouse cortex was dramatically decreased (Figure 3G–I). To determine whether H3K27me3 regulates PV expression, H3K27me3 chromatin immunoprecipitation (ChIP) assays by five distinct primer pairs targeting the *pvalb* promoter locus were carried out (Figure 3J). We observed robust H3K27me3 in the *pvalb* promoter region from −1349 to −513 in WT cortical neurons, whereas the H3K27me3 occupancy at the *pvalb* promoter locus was dramatically decreased in *Men1*‐knockout neurons (Figure 3K). Furthermore, Menin‐ChIP assays showed a direct binding of Menin to the *pvalb* promoter region (Figure 3L; S‐Figure S10, Supporting Information). Together, our data suggest that Menin binds to *pvalb* promoter region and consequently facilitates chromatin remodeling for *pvalb* transcription.

### 2.4 Menin Restoration, PV Downregulation or PV Neuronal Activity Inhibition in PV Interneurons All Rescue Depressive‐Like Behaviors in *Men1*‐PcKO Mice

To further confirm that the Menin expression in PV interneurons determines the depressive‐like behaviors, exogenous Menin was expressed specifically in PV interneurons by injecting Cre recombinase‐dependent Menin‐AAV into the ventricle of newborn *Men1*‐PcKO (P0) mice. Behavioral tests were subsequently performed 60 days after injection (Figure 4A). The restoration expressions of Menin in PV interneurons were first confirmed by immunostaining (Figure 4B). Menin restoration significantly rescued depressive‐like behaviors in TST, FST and SPT tests (Figure 4C–E), as well as reduced PV expression measured by immunofluorescence staining, western and RT‐PCR analyses (S‐Figure S11A–E, Supporting Information). To investigate whether the increasing of PV is related with the depressive‐like behaviors in PcKO mice, PV expression was down‐regulated by bilateral injecting Cre recombinase‐dependent shRNA *pvalb* AAVs (with EGFP expression tag): AAV‐EGFP‐shRNA(*pvalb*) into the mPFC area of *Men1*‐PcKO mice (Figure 4F). The knockdown efficiency of PV was measured by immunostaining and RT‐PCR (Figure 4G–I). Significantly, the depressive‐like behaviors in PcKO mice were efficiently rescued by PV downregulation (Figure 4J–M).

We next wondered whether the PV neuronal activity is related with depressive phenotype of PcKO mice. We used chemogenetic approach using CNO‐activated designer receptors exclusively activated by designer drugs (Gq‐DREADDs) to inactivate of PV interneurons. The AAV9‐hSyn‐DIO‐hM4D(Gi) or control AAV9‐hSyn‐DIO‐mCherry AAVs were injected into the mPFC area of PV cre or *Men1*‐PcKO mice (Figure 4N).^[^ ^24^ ^]^ The cFos staining were performed to confirm the inactivation of PV interneurons (the overlap of cFos with mCherry) (S‐Figure S11F,G, Supporting Information). These mice were subjected to depressive behaviors tests. Notably, the depressive‐like behaviors of PcKO mice were significantly rescued by inhibition of PV neuronal activity (Figure 4O–R). The activity inhibition of PV interneurons also reduced the expression of PV without affecting Menin expression (Figure 4S,T; S‐Figure S11H,I, Supporting Information).

The effects of Menin on the synaptic activity of PV interneurons were further investigated. Following the strategy as descripted in Figure 4U, the Cre recombinase‐dependent *MEN1*‐AAV or control AAV was injected into the mPFC of *Men1*‐PcKO mice or PV cre mice. Electrophysiology recording was performed in EGFP‐labeled PV interneurons from *PV cre*+AAV‐GFP, *PcKO*+AAV‐GFP and *PcKO*+AAV‐GFP‐*MEN1* brain slices (Figure 4U). We observed that the AP threshold levels are lower in GFP labeled PV interneurons of *PcKO* mice compare to *PV cre* mice, suggesting increased excitability in PV interneurons of *PcKO*+AAV‐GFP mice. Significantly, the restoration Menin expression in PV interneurons of *PcKO* mice (*PcKO*+AAV‐GFP‐*MEN1*) decreased the excitability of PV interneuron (Figure 4V–X). Besides, we also observed the increased frequency of mIPSC in PV interneurons of *PcKO*+AAV‐GFP mice compared to *PV cre*+AAV‐GFP mice, while Menin restoration lowed this increasing (Figure 4Y,Z). The amplitude and frequency of mEPSC were not affected.

The above results indicated that re‐expression of Menin or inhibition PV expression/PV interneuron activity all could rescue the depressive behaviors.

### 2.5 The Antidepressant Effect of Ketamine Requires Menin

Ketamine has been found to produce an antidepressant effect at sub‐anesthetic doses.^[^ ^25^ ^]^ We next determine whether the depressive behaviors resulting from Menin deficiency can be ameliorated by ketamine. As expected, in the LPS‐induced depressive mouse model, ketamine effectively attenuated the depressive behaviors (Figure 5A–E) and decreased the PV expression (Figure 5F,H,J). However, ketamine failed to ameliorate the depressive behaviors in *Men1*‐PcKO mice (Figure 5A–E), and that the PV expression remained unchanged (Figure 5G,I,K). More importantly, the double immunostaining of PV and Menin revealed that Menin expressions decreased in LPS induced depressive mouse brain, and ketamine administration recovered the Menin expression (Figure 5L,M). In contrary, fluoxetine, a selective serotonin reuptake inhibitor (SSRI), attenuated the depressive behaviors in *Men1*‐PcKO mice (Figure 5N–R). These results suggest that the presence of Menin is required for the anti‐depression effects of ketamine.

### 2.6 Ketamine Stabilizes Menin Through Inhibiting PKA‐Mediated Phosphorylation

The above data suggested that the anti‐depressant effect of ketamine is partially through the up‐regulation of Menin expression. We found that ketamine administration significantly increased Menin protein levels at a time and dose‐dependent manner, without affecting its mRNA levels, both in vitro and in vivo (Figure 6A–F). Cycloheximide chase assay further indicated that ketamine significantly improved the protein stability of Menin (Figure 6G,H), suggesting that ketamine may modulate the post‐translational modification of Menin to regulate its protein stability. Menin has been reported to be phosphorylated by protein kinase A (PKA) at Ser 487 residue.^[^ ^26^ ^]^ We found that the Menin protein levels were reduced by the PKA activator forskolin treatment, however, increased by the PKA inhibitor H‐89 treatments in primary cultured neurons (Figure 6I,J). When serine 487 of Menin was mutated to non‐phosphorylated status (S487A) or phosphorylated status (S487D), the degradation rate of Menin was significantly reduced or accelerated, respectively (Figure 6K,L). Finally, we found that the kinase activity of PKA was significantly inhibited by ketamine (Figure 6M,N). These data strongly indicate that phosphorylation of residue S487 by PKA is important for the stability of Menin, which can be modulated by ketamine.

## 3 Discussion

The present study uncovered that Menin deficiency in PV interneurons leads to depressive behavior in mice and inhibits the PV transcription by H3K27me3 modification. We also revealed that ketamine requires Menin to exert its antidepressant effect by stabilizing Menin protein stability. These results suggested for the importance of Menin in regulating PV expression, the pathogenesis of depression, and maintaining the antidepressant activity of ketamine.

GABA is the main inhibitory neurotransmitter for interneurons, which regulates a variety of partial neurotransmitter systems, especially the glutamatergic excitatory counterpart.^[^ ^27^ ^]^ The dysregulation of GABAergic system in PFC may lead to abnormal behaviors and synaptic responses, including dendrite reorganization of interneurons and changes of electrophysiological responses,^[^ ^28^ ^]^ while the consequence of GABAergic dysfunction remains controversial. Low GABAergic tone was reported in the central nervous system of depression,^[^ ^14^, ^29^ ^]^ however, there is a controversy in therapeutic outcomes.^[^ ^30^ ^]^ As the most abundant interneuron, PV interneurons are tightly involved in depression, whether the PV level is directly related with the pathogenesis of depression is still unknow.

Previous studies and work from our group showed that PV expression was increased in the brain of the CUMS‐ or LPS‐induced depressed mice,^[^ ^17^, ^31^ ^]^ suggesting a potential mechanism by which chronic stress leads to PV increase. Given that PV expression in a specific subtype of GABAergic neurons, UCMS treatment has also been shown to increase the expression of GABA receptor α1.^[^ ^32^ ^]^ The expression of α5 GABA A receptor in the parietal cortex from depressed patients is higher than that of control subjects.^[^ ^33^ ^]^ Chronic stress increases inhibition of infralimbic cortex (ilPFC) glutamatergic output neurons via an increase in GABA release, likely owing to increased GABAergic innervation of glutamatergic output neurons.^[^ ^16a^ ^]^ As a unique depressive mouse model, Menin‐G503D mice exhibit increased GABAergic functions. In consistent with above, we observed that the expression of PV, but not SST and VIP was markedly increased in G503D cortices. CUMS and *Men1*‐PcKO mouse brains also showed increased PV expression. The PV expression levels in the brain have also been reported to correlate with neuronal plasticity and psychiatric illnesses. Enrichment environments decrease PV and GAD67 expression, promoting the configuration of “low‐PV‐network” that enhances structural synaptic plasticity and memory consolidation. In contrary, stress such as fear condition treated mice exhibits higher PV and GAD67 expression, resulting a “high‐PV‐network” that impair the synaptic plasticity.^[^ ^34^ ^]^ The increased proportion of high PV (in other words, increased expression of PV) is closely related to many mental diseases.^[^ ^35^ ^]^ Thus, we speculate that the upregulation of PV expressions in depressive mice may weaken PV neuronal function and impair plasticity, in consequence, affects the neuronal network.

The functions of PV interneurons have been widely researched, but the PV expression regulating mechanism is hardly known. Since little is known about the correlation between PV expression levels with the function of PV^+^ neurons. One main reason that restricted this research is the lack of other makers for PV interneuron, except PV. Thus, PV expression is the only standard to evaluate the PV^+^ cells. In our study, we found that Menin deficiency affects PV protein expression not only in PV neurons but also in other type of neurons that nearly express PV in normal conditions (S‐Figure S8, Supporting Information). This raises a new question to how to define or classify neuronal types more accurately. In depression, the increased PV expression may lead to re‐classification of neurons, caused by the expression of PV in “non‐PV” cells. The changes in PV expression may significantly affect the normal function of the neuron, or may change the electrophysiological characteristics of the neuron, since it is no longer the original neuron type.

The variation of PV levels plays key role in maintaining neuronal network, however, the mechanism underlying PV expression regulation remains unclear.^[^ ^36^ ^]^ Our results demonstrate that levels of histone methylation (H3K27me3) were decreased in PV^+^ interneurons of *Men1* deficient mice, suggesting a role of Menin in remodeling the chromatin complex. Interestingly, the H3K27me3 occupancy at the *pvalb* promoter locus was also dramatically decreased in *Men1*‐knockout neurons. Menin is well‐known to regulate gene expression by interacting with histone modifiers, such as H3K4 methyltransferases^[^ ^12^ ^]^ and H3K27 methyltransferases^[^ ^14^ ^]^ in cancer tissues. However, its epigenetic regulation is little known in neurons, especially in interneurons. The current data provide strong support for its epigenetic regulation on *pvalb* transcription in embryonic and adult stages.

As an NMDA receptor inhibitor and an antagonist of glutamatergic neurons, ketamine also inhibits GABAergic neurons, which may affect principle‐glutamatergic neuronal activity. It has been shown that ketamine regulates the spontaneous firing of GABAergic interneurons to a greater extent than of pyramidal neurons.^[^ ^20a^ ^]^ Indeed, the absence of Menin in inhibitory neurons results in increased GABAergic synaptic transmission, which coincides with blocked enhancement of excitatory synaptic potentials by ketamine. Therefore, ketamine increases expression of Menin in neurons that are central in maintaining the chronic (in a range of days) potentiation of excitatory synaptic transmission. Consistent with our current finding, ketamine has also been reported to attenuate depressive behaviors by reducing PV expression in mice.^[^ ^37^ ^]^ Moreover, apocynin, a nicotinamide adenine dinucleotide phosphate hydrogen (NADPH) oxidase inhibitor, can block the antidepressant‐effects and reduction in PV levels induced by ketamine,^[^ ^38^ ^]^ further supporting the effect of increased expression of PV on the occurrence of depression.

The fact that *Men1*‐PcKO mice did not respond to ketamine treatment indicates that the antidepressant effect of ketamine is dependent on the presence of Menin. We demonstrate that ketamine stabilizes Menin protein by inhibiting of protein kinase A (PKA).^[^ ^39^ ^]^ Ketamine has various ways to achieve its antidepressant effect by regulating the activity of calcium/calmodulin kinase II, protein kinase D and PKA,^[^ ^19^, ^40^ ^]^ but their downstream molecular mechanism is unclear. Our finding further supported that ketamine is also capable to directly regulate protein expression to exert its anti‐depressant actions.^[^ ^19^, ^40^ ^]^

We previously found that astrocytic Menin deletion leads to depression in mice.^[^ ^8^ ^]^ The deletion of Menin in astrocyte up‐regulated neuroinflammatory processes by NF‐κB pathway. We also identified a SNP, rs375804228, in human *MEN1*, where G503D substitution is associated with a higher risk of MDD onset. Here, we created a mouse model with the point mutation (G503D) at *Men1* locus. The Menin (G503D) mice specifically exhibit depressive‐like behaviors. As predicted, the neuroinflammation is increased in the brain from Menin (G503D) mice. Meantime, we also observed PV interneurons dysfunction in these mice. As co‐transcription factor, Menin plays distinguished roles in astrocyte and interneurons. The lost function of Menin accelerates the inflammation in astrocyte but down‐regulation of PV in PV neurons. However, we found that there is no significant difference in the inflammatory levels in PcKO mice compared with control mice, which may suggest that these two antidepressant mechanisms of Menin might not be closely relevant. The relationship between the two paths to depression led by Menin deficiency, the inflamed astrocyte, and hyperactive PV interneurons, needs further exploration.

In summary, by examining several Menin mutated and neuronal conditional knockout mouse models, we demonstrate that Menin‐mediated PV expression is involved in the pathogenesis of depression in mice. Our data suggest that the anti‐depressive effect of ketamine is at least partially by altering PV expression through Menin. The current data also provide inspiration for precision application of ketamine in MDD patients with *MEN1* mutation. Furthermore, our results also suggested that pharmacological manipulation of GABA signaling is a potential treatment strategy for depression disorders due to mutations in chromatin remodeling genes.

## 4 Experimental Section

### Animals

All mice were maintained within the laboratory animal center at Xiamen University, and all experimental procedures involved were performed according to protocols approved by the Institutional Animal Care and Use Committee at Xiamen University. The approval number of the protocol is XMULAC20200050. This study also abides by the provisions of the Biosafety Law of the People's Republic of China, the Regulations on the Administration of Experimental Animals, the National Standards for Experimental Animals (GB14925‐2010), the Guidelines for Ethical Review of the Welfare of Experimental Animals (GBT 35892–2018), and the relevant rules and regulations formulated by Xiamen University. Mice were housed under a 12 h light/dark cycle with free access to standard rodent chow and water. Each cage housed a maximum of four mice. Mice were maintained under specific‐pathogen‐free SPF conditions and were not subject to immune suppression. Health of the animals used was regularly controlled by animal caretakers. All mice used in the study were not previously involved in other experimental procedures, and were drug/test naïve. A mixture of 2‐month‐old litter/age‐matched male and female mice were employed in all studies and no differences between sexes were observed. Animals were used according to “3Rs” principles (Replacement, Reduction and Refinement) in all experimental procedures.

Menin (G503D) Knock‐in mice were created as a C57BL/6 mouse model with point mutation (G503D) at mouse *Men1* locus by CRISPR/Cas‐mediated genome engineering from Cyagen Biosciences (Suzhou, China). The G503 was located on exon 10, which was selected as target site. gRNA targeting vector and donor oligo (with targeting sequence, flanked by 120 bp homologous sequences combined on both sides). The G503D (GGC to GAC) mutation sites in donor oligo were introduced into exon 10 by homology‐directed repair. Cas9 mRNA, gRNA generated by in vitro transcription and donor oligo were co‐injected into fertilized eggs for KI mouse production. The pups will be genotyped by PCR followed by sequence analysis. Genomic region of mouse *Men1* locus is diagrammed in S‐Figure 1A (gene is oriented from left to right; total size was 5.88 kb). Solid bars represent ORF; open bars present UTRs. gRNA1 (matches forward strand of gene): TTGGACAAGGGCCCGGGCTCAGG. Donor oligo sequence:CCCCGAAGAGAGTCCAAGCCTGAGGAGCCACCACCACCCAAGAAGCCTGCATTGGACAAGGACCCGGGCTCAGGACAAAGTGCAGGGTCGGGACCACCTAGGAAAACGTCAGGGACTGTCCCA. The target region of mouse *Men1* locus was amplified by PCR with specific primers.

The floxed *Men1*mouse strain (*Men1* ^F/F^) was provided by Dr. Guanghui Jin and Dr. Xianxin Hua.^[^ ^41^ ^]^ *Nestin‐Cre*, *PV‐Cre*, and *SST‐Cre* transgenic mice^[^ ^42^ ^]^ were obtained from Dr. Jiawei Zhou at the Institute of Neuroscience, Chinese Academy of Science; these mice are available from Jackson Laboratory. *Men1*‐NcKO, PcKO, and SckO mice were obtained by crossing *Men1* ^F/F^ mice with respective *Nestin‐Cre*, *PV‐Cre or SST‐Cre* mouse lines. *Men1* ^F/F^ mice were used as controls.

### LPS‐Induced Mouse Model

Male C57BL6/J mice (2 months) were intraperitoneally injected with LPS (Sigma, L‐2880) dissolved in sterile 0.9% saline at 0.5 mg kg^−1^. This dosage was used to stimulate infection without inducing obvious inflammation or other maladies. Saline or LPS injection was administered between 09:00 and 09:30 a.m. daily for 10 days. Behavioral tests were performed 24 h following the last injection. Brain tissue and serum was dissected/sampled 24 h following behavioral testing.

### Chronic Unpredictable Mild Stress (CUMS)

C57BL/6 wild‐type mice were divided into control and CUMS groups. CUMS mice were maintained in individual cages. CUMS involved exposure to a variety of mild stressors: i) 24 h food deprivation, ii) 24 h water deprivation, iii) 1 h of exposure to an empty bottle, iv) 7 h cage tilt (45^o^), v) overnight illumination, vi) 24 h habitation in a soiled cage (200 mL of water in 100 g of saw dust bedding), vii) 30 min of forced swimming at 8 °C, viii) 2 h of physical restraint, and ix) 24 h of exposure to a foreign object (e.g., a piece of plastic). These stressors were randomly scheduled over a 3 weeks period.

### Experimental Design

All experiments described in this study were performed a minimum of three biological repetition or three independent experiments. A statistical method was not used to predetermine sample size. The sample size per experiment was determined according to previous publications. All the experiments involving mice experiments were performed randomly and analyzed in a blind manner. Specifically, mouse genotype was de‐identified during the experimental trials in the behavioral analyses described. Similarly, mice subjected to electrophysiological analysis were identified by number where the genotype was withheld during electrophysiological recording. In the image analysis for neuronal morphology, images were acquired and genotype/treatment was de‐identified in the image files for analysis. No data was excluded from the analysis.

### Stereotactic Injection of Adeno‐Associated Virus

pAAV‐EF1α‐Dio‐GFP‐WPRE (virus titer: 5.25 × 10^12^ mL^−1^), pAAV‐EF1α‐Dio‐*MEN1*‐GFP‐WPRE (virus titer: 6.29 × 10^12^ mL^−1^), rAAV‐EF1α‐Dio‐*pvalb*‐P2A‐EGFP‐WPRE (virus titer: 5.15 × 10^12^ L^−1^), rAAV‐EF1α‐Dio‐EGFP‐WPRE (virus titer: 5.05 × 10^12^ mL^−1^), pAAV‐CMV‐Dio‐EGFP‐Scramble‐WPRE (virus titer: 5.11 × 10^12^ mL^−1^) and pAAV‐CMV‐Dio‐EGFP‐shRNA(*pvalb*)‐WPRE (virus titer: 5.33 × 10^12^ mL^−1^) were purchased from BrainVTA (Wuhan, China). Packaged viruses (pAAV‐EF1α‐Dio‐GFP‐WPRE and pAAV‐EF1α‐Dio‐*MEN1*‐GFP‐WPRE) were stereotactically injected into the bilateral ventricles of control mice or PcKO mice. The injection site was located two‐fifths of the distance along a line defined between each eye and the lambda intersection of the skull. To confirm region‐specific overexpression of *Men1* in mouse brains, mice were anesthetized and sacrificed 2 months after injection, whereupon brain tissues were rapidly removed and analyzed using histological immunofluorescence staining. Packaged viruses (rAAV‐EF1α‐Dio‐*pvalb*‐P2A‐EGFP‐WPRE, rAAV‐EF1α‐Dio‐EGFP‐WPRE, pAAV‐CMV‐Dio‐EGFP‐Scramble‐WPRE and pAAV‐CMV‐Dio‐EGFP‐shRNA(*pvalb*)‐WPRE) were injected bilaterally into the mPFC (AP: +1.7, ML: +/− 0.4, DV: − 2.5 mm) of control mice or PcKO mice over the course of 5 min with an additional 15‐min wait to allow for diffusion.

For chemogenetic activation or inhibition of PV interneuron in mPFC area, adult male mice were anesthetized with isoflurane (4% induction, 1.5–2% maintenance) and placed in the stereotaxic device (Stoelting, Wood Dale, IL). Nanoliters of 250–500 of AAV9‐hSyn‐DIO‐hM4D(Gi)‐mCherry, a viral vector leading to Cre‐dependent expression of a silencing DREADD receptor in PV‐expressing neurons, or the control virus AAV9‐hSyn‐DIO‐mCherry (Addgene, Watertown, MA) were injected bilaterally into the mPFC (AP: +1.7, ML: +/− 0.4, DV: − 2.5 mm) over the course of 5 min with an additional 15 min wait to allow for diffusion. CNO was administered via a single IP injection (5 mg kg^−1^) and animals were then perfused 30 min before the behavior tests.

### Statistics

All data presented were expressed as arithmetic mean±SEM. All statistical analyses were performed using GraphPad Prism version 5.0. Null hypotheses were rejected at p values equal to or >0.05. For statistical comparisons between two groups, a Shapiro‐Wilk normality test (Prism) was first performed to determine whether the data was likely normally distributed. For normally distributed data, unpaired Student's *t*‐tests were used to evaluate statistical significance of differences between the two groups. Statistically significant differences between groups were determined using one‐way ANOVA. In evaluating multiple comparisons, Bonferroni(≤4 groups)/Tukey's correction(>4 groups)methods were used to adjust p values accordingly to lower the probability of type I errors. All electrophysiological results were analyzed using Sigma Stat 4 statistical software. Statistical significance was evaluated by one‐way ANOVA with Holm‐Sidak pair‐wise tests. Values of *p* < 0.05 were considered statistically significant. DNASTAR Laser gene software (version 7.1) was used to analyze Sanger sequencing data. For behavior tests described in the study, the number of mice analyzed in each group are described in the respective figure legends (Figures 1, 2, 4, and 5); the number of slices and mice used for electrophysiological recordings is defined. For other experiments (Western Blotting, Immunofluorescence, and Electrophysiology), the definition of “n” in the context are described in respective figure legends. All statistical details, including the exact value of *n*, what *n* represents, and which statistical test was performed, is described in the figure legends.

## Conflict of Interest

The authors declare no conflict of interest.

## Author Contributions

L.L. and K.Z. contributed equally to this work. L.L. and J.Z. performed conceptualization. L.L, K.Z, H.L., C.H, H.L. prepared and maintained the mice. L.L., K.Z., and Z.C. designed and performed morphological analysis and biochemical assays. L.L., H.L., H.W., G.C. performed behavior tests. M.W. and H.S. performed electrophysiology experiments. L.L. and J.Z. wrote original manuscript. L.L., J.Z., H.C., Z.C., H.X., T.F., and Q.X. wrote review and performed editing. Q.X. performed *MEN1* SNP screening. J.Z. performed supervision. All authors reviewed and gave final approval to the manuscript.

## Supporting information

Supporting Information

Supplemental Dataset 1

Supplemental Dataset 2

## Data Availability

The data that support the findings of this study are available from the corresponding author upon reasonable request.
