# Supplementary material for: Menin Reduces Parvalbumin Expression and is Required for the Anti‐Depressant Function of Ketamine
Source: Adv Sci (Weinh). 2023 Dec 3;11(5):2305659. doi: 10.1002/advs.202305659 (PMC10837338; doi:10.1002/advs.202305659)
Supplement: Supplementary file 1 — Supporting Information [file ADVS-11-2305659-s003.pdf]

ADVANCED SCIENCE
Open Access

Supporting Information

for *Adv. Sci.*, DOI 10.1002/advs.202305659

Menin Reduces Parvalbumin Expression and is Required for the Anti-Depressant Function of Ketamine

Lige Leng, Kai Zhuang, Hui Lin, Jinjun Ding, Shangchen Yang, Ziqi Yuan, Changquan Huang, Guimiao Chen, Zhenlei Chen, Mengdan Wang, Han Wang, Hao Sun, Huifang Li, He Chang, Zhenyi Chen, Qi Xu, Tifei Yuan and Jie Zhang**

Supplementary Information for

Menin reduces parvalbumin expression and is required for the anti-depressant function of ketamine

Lige Leng *et al.*

*Corresponding author: Jie Zhang, E-mail: jiezhang@xmu.edu.cn; Lige Leng, E-mail: lenglige@xmu.edu.cn.

Inventory of Supplemental Information:

Supplementary Text of Materials and Methods
Supplementary Dataset 1, 2
Figures S1 to S11
- Figure S1, related to Figure 1
- Figure S2, related to Figure 1
- Figure S3, related to Figure 1
- Figure S4, related to Figure 1
- Figure S5, related to Figure 2
- Figure S6, related to Figure 2
- Figure S7, related to Figure 2
- Figure S8, related to Figure 2
- Figure S9, related to Figure 2
- Figure S10, related to Figure 3
- Figure S11, related to Figure 4

Supplementary Text

Materials and Methods

Neuron and HEK293T culture procedures

Primary neurons were dissected from timed-pregnant females at E16.5. Briefly, brain cortices were dissected from pups of varying genotypes. The meninges were removed, and cortical tissue was dissociated by enzymatic digestion. Isolated primary neurons were plated on poly-D-lysine coated dishes, and cultured in Neurobasal medium supplemented with B27 (Gibco)/1% penicillin/streptomycin (Invitrogen) and

maintained in a 5% CO_2 incubator at 37°C. HEK293T was purchased from ATCC for a vial (2×10^6)(Cat#CRL-3216, RRID:CVCL_0063). HEK293T was cultured in DMEM supplemented with 10% FBS and maintained at 37°C in humidified air containing 5% CO_2.

ChIP

ChIP procedures were performed followed the manufacturer's instructions (17-295; Millipore). Briefly, primary neurons from control and NcKO mice (dissected at E16.5) were cultured for 10–12 days and fixed for 10 min at room temperature with media containing 1% formaldehyde and quenched with 125 mM glycine for 5 min. Fixed homogenates were washed twice using ice-cold PBS containing protease inhibitors. Fixed nuclei were pelleted at 4 min at 2,000 rpm and re-suspended in SDS Lysis Buffer (catalog #20-163), where chromatin was sheared using a SCIENTZ ultrasonic apparatus set to 28% power for 14 cycles of a 4.5 s sonication and a 9.0 s resting stage on ice. The sonicated cell supernatant was diluted 10-fold in ChIP dilution buffer (catalog #20-153) and pre-cleared using protein an Agarose/Salomon Sperm DNA (catalog #16-157). After brief centrifugation, ChIP was performed using 3 mg Menin antibody (A300-105A; Bethyl), H3K27me3(Ab8580; Abcam) or normal rabbit IgG (H2615; Santa Cruz) antibody incubated overnight, followed by enrichment using protein A Sepharose beads for 4 hr. Beads were washed three times with four different buffers (low-salt immune complex wash buffer, high-salt immune complex wash buffer, and LiCl immune complex wash buffer) and one wash with TE (50 mM Tris HCl, 10 mM EDTA). Chromatin was eluted by agitation at 65°C for 20 min in TES (TE plus 1%

SDS) and reverse crosslinked overnight at 65°C.Chromatin was subjected to RNase and Proteinase K treatment, followed by DNA purification by phenol chloroform extraction and ethanol precipitation. DNA pellets were re-suspended in 10 mM Tris and subjected to qPCR (480; Roche).

Electrophysiological recording

Whole-cell patch clamp recordings were obtained from pyramidal neurons in the cortex of G503D mice and littermate control mice; NcKO and littermate control mice using 4–8 MΩ borosilicate glass pipettes (Harvard Apparatus) containing (in mmol 1^{-1}): 8.0 NaCl, nominally 0.0001 $CaCl_2$ (without Ca^{2+} buffering, the concentration cannot be precise), 10.0 Na-Hepes, 130 potassium gluconate, 1.0 $MgCl_2$, 0.3 NaGTP, and 2.0 NaATP (pH adjusted to 7.4 with methanesulfonic acid; 295–300 mosmol 1^{-1}), unless otherwise specified. Glass pipettes with an Ag–AgCl electrode were connected to a CV-4 headstage and Axopatch-1D amplifier with a Digidata 1200 interface (Axon Instruments) and positioned within the tissue using a motorized patch-clamp micromanipulator. Cell-attached 4–10 GΩ seals were obtained using the blind-patch technique as described elsewhere. Upon seal formation, the whole-cell patch configuration was achieved by gently applying brief suction. Typical whole-cell access resistance (R_a) was 5–30 MΩ and whole-cell leak was below 20 pA. R_a and leak were checked prior to each experiment and if *R*a or leak changed by more than 20% or 30 pA, respectively, during a recording then the recording was discarded. Miniature excitatory postsynaptic currents (mEPSCs) and miniature inhibitory postsynaptic currents (mIPSCs) were recorded in the presence of tetrodotoxin (500 nmol L^{-1}). We recorded

mEPSCs and mIPSCs at holding potentials of –70 and 0 mV, respectively, in the same cell (3 minutes each; n＞20cells/group).

RNA-Sequencing analysis

Cortex of G503D mice and littermate control mice were isolated to obtain RNA which was then used for RNA sequencing analysis. cDNA library construction and sequencing were performed by the Beijing Genomics Institute using Illuminate platform. High-quality reads were aligned to the mouse reference genome using Bowtie 2. Expression levels for each of the genes were normalized to fragments per kilobase of exon model per million mapped reads (FPKM) using RNA-seq by Expectation Maximization (RSEM). Genes with ≥ 2-fold change and $P<0.05$ were considered statistically significant.

PcKO and control mice were crossed with Ai14 TD tomato reporter mice to obtain 1-month-old off spring mice whose PV neurons with fluorescence. After washing, the freshly peeled brain tissue of the mice was cut into 8 sagittal sections and transferred into the gentle Macs C tube (Meltenyi Biotech). Single cell suspensions of the above mice brain were separated according to the manufacturer's instructions. Then flow cytometry was used to isolate PV neurons of PcKO mice and control mice. Isolated RNA was subsequently used for RNA-seq analysis. cDNA library construction and sequencing were performed by the Beijing Genomics Institute using BGISEQ-500 platform. High-quality reads were aligned to the mouse reference genome using Bowtie 2. Expression levels for each of the genes were normalized to fragments per kilobase of exon model per million mapped reads (FPKM) using RNA-seq by Expectation

Maximization (RSEM). We identified DEGs (differential expressed genes) between samples and performed clustering analysis and functional annotation. Genes with ≥ 2-fold change and P<0.05 were considered statistically significant. Pathways overrepresented by DEGs were annotated in the KEGG (Kyoto Encyclopedia of Genes and Genomes) database.

Behavioral Studies

Forced Swimming Test

The forced swim test (FST) was used to assess depressive-like behavior. Mice were placed in a container filled with water that eventually resulted in immobility, reflecting behavioral despair. Water (23 ± 1°C) was placed in a transparent acrylic cylinder bath (10cm in diameter, 20cm in height) filled to a depth of 13cm. Mice were placed in the water for six minutes using a video tracking system (Smart 3.0). Immobility duration (%) within the final 5 minutes of testing was recorded.

Tail Suspension Test

The tail-suspension test was used to assess the efficacy of antidepressants in mice. Mice were suspended by their tails from an acrylic bar (15cm in diameter, 30cm in height) for six minutes and monitored using a video tracking system (Smart 3.0). Escape-related behavior was assessed, where immobility duration (%) during the 6 minutes suspension period was recorded.

Sucrose Preference Test (SPT) and Sucrose Consumption Test (SCT)

Animals were first trained to consume a 1% sucrose solution from two differing bottles (48 hours before the formal experiment). Twenty-four hours later, the animals

were allowed free access to 1% sucrose and water from two differing bottles. To avoid bottle side preference, the two bottles were switched once. The amounts in the two bottles were measured after 24 hours and sucrose preference was calculated according to the following formula: sucrose preferences (%) = sucrose consumption / (sucrose + water consumption) ×100%.

Morris Water Maze

The water maze used in this study comprised a circular tank 120 cm in diameter with a platform filled with tap water at a temperature of 22 ± 2°C. Different shapes were posted along the walls of the tank, which served as spatial reference cues. A camera was mounted above the maze to record the swimming traces in the water maze. During the acquisition trials, the platform was submerged 1-2 cm below the water surface, mice were place into the maze at one of four points (N, S, E, W) facing the wall of the tank. Mice were allowed to search for a platform for 60 s. If a mouse failed to find the platform, it was guided to the platform and maintained on the platform for 10 s. Four trials a day were conducted with at an intermission of 1 h minimum between trials. Escape latency which indicated spatial learning memory acquisition, was recorded for each trial. On day 7, the platform was removed and a probe test was conducted. The percentage time spent in each of the four quadrants and the number of target (platform) area crossings, mean speed, total distance was recorded.

Rotarod Test

Mice were placed on a stationary rotarod (AccuRotor Rota Rod Tall Unit, 63-cm fall height, 30-mm diameter rotating dowel; Accuscan, Columbus, OH). The dowel was

then accelerated to 60 rpm min^{-1}, and the latency to fall (in seconds) was recorded. The procedure was repeated over 4 consecutive trials, which was averaged to yield the daily latency to fall for each mouse. If an animal fell off the rotarod rapidly (e.g., due to inattention or slips), they were placed back on the rotarod for an additional trial, and the latency was not included in the daily average. The entire procedure was repeated for 2 additional days for a total of 3 days. In addition to the average latency across the 4 trials per day, the maximum latency to fall per day was also analyzed.

Open Field Test

To explore locomotion and spontaneous activity, we characterized mouse behavior as they freely explored an open-field plastic chamber (40-cm width ×40-cm length × 30-cm height) using a video tracking system (Smart 3.0). 2-months old mice were placed in this arena for 10 min., and total distance and time spent in the center region (20cm×20cm) was recorded.

Elevated Plus Maze

The apparatus comprised two opposing open arms (50×10 cm) and two opposing closed arms with roofless gray walls (40 cm) connected by a central square platform and positioned 50 cm above the ground. Mice were placed in the open arms facing an open arm, and their behavior was tracked for 5 min with an overhead camera and Smart 3.0 software. Time spent in the open arms (%) was reported.

Western blotting

Cultured cells and mouse brain tissues (include cortex and hippocampus) were homogenized in lysis buffer (RIPA) on ice for 40 min, and subsequently centrifuged at

12,000 rpm for 10 min at 4°C. Sample containing 30 g of protein was separated using 10% SDS-PAGE gels. Proteins were transferred onto PVDF (polyvinylidene difluoride) membranes in an ice-cold buffer (25 mM TrisHCl, 192 mM glycine, and 20% methanol) by electro transfer for 1.5 h. Immunoblots were probed with indicated antibodies. Goat-anti secondary antibodies were purchased from Millipore (#AP132P, #AP124P). Quantification of band intensities were normalized to β-actin or GAPDH.

Quantitative RT-PCR

Total RNA from animal tissues and cells were isolated using Trizol reagent according to the manufacturer's instructions (Invitrogen). Reverse transcription was performed using ReverTra Ace qPCR RT Master Mix (Toyobo, FSQ-201). RNA concentrations were adjusted to 1.2 μg μL^{-1} in nuclease-free water, and total RNA was reverse-transcribed in a 20 μL reaction volume. cDNA was amplified by real-time quantitative RT-PCR using SYBR Green (Roche) reagent. Samples were assayed in triplicate and GAPDH was used as an internal control. Primer sequences used in this study are as follows:

Gene	Primer Sequences	
Men1	Forward	5'-ACCTATCCATCATTGCTGCCCTCT-3'
	Reverse	5'-ACCAGTTCGCGACTAGAAACACCT-3'
Gapdh	Forward	5'-AGGTCGGTGTGAACGGATTTG-3'
	Reverse	5'-TGTAGACCATGTAGTTGAGGTCA-3'
Pvalb	Forward	5'-ATCAAGAAGGCGATAGGAGCC-3'
	Reverse	5'-GGCCAGAAGCGTCTTTGTT-3'
Gad1	Forward	5'-CACAGGTCACCCTCGATTTTT-3'
	Reverse	5'-ACCATCCAACGATCTCTCTCATC-3'
Gad2	Forward	5'-TCCGGCTTTTGGTCCTTCG-3'
	Reverse	5'-ATGCCGCCCGTGAACTTTT-3'
SST	Forward	5'-ACCGGGAAACAGGAACTGG-3'
	Reverse	5'-TTGCTGGGTTCGAGTTGGC-3'
VIP	Forward	5'-AGTGTGCTGTTCTCTCAGTCG-3'

	Reverse	5'-GCCATTTTCTGCTAAGGGATTCT-3'
IL-1β	Forward	5'-ATGGCAACTGTTCCTGAACTCAACT-3'
	Reverse	5'-CAGGACAGGTATAGATTCTTTCCTTT-3'
IL-6	Forward	5'-TCCAGTTGCCTTCTTGGGAC-3'
	Reverse	5'-GTGTAATTAAGCCTCCGACTTG-3'
IL-2	Forward	5'-CGCACCCACTTCAAGCTCCACTTC-3'
	Reverse	5'-ATTCTGTGGCCTGCTTGGGCAAG-3'
TGF β	Forward	5'-TTCTGTCTACTGAACTTCGGGGTGATCGGTCC-3'
	Reverse	5'-GTATGAGATAGCAAATCGGCTGACGGTGTGGG-3'
Cox-2	Forward	5'-CCCTGCTGCCCGACACCTTC-3'
	Reverse	5'-CCAGCAACCCGGCCAGCAAT-3'
Pvalb promoter-1	Forward	5'- AGCTACTCTGAGCCTTATTCC-3'
(-1965~ -1749)	Reverse	5'- ATCTCATCAAGGCAGACTGAA-3'
Pvalb promoter-2	Forward	5'- CATCCAAGGATCCAGAGCGT-3'
(-1349~ -1169)	Reverse	5'- AGCCTCATTTAGCTGCTTCAC-3'
Pvalb promoter-3	Forward	5'- CCAAAGCACCCTTACCAGTGA-3'
(-675~ -513)	Reverse	5'- AGCTTCCAGGCAATATGTCAG-3'
Pvalb promoter-4	Forward	5'- TGTGCCCTGCTTGGACCTTA-3'
(-282~ -158)	Reverse	5'- TTCCGGTGTCAGGTACTCCC-3'
Pvalb promoter-5	Forward	5'- CTGAGTTCTCTCCGAGCTTCT-3'
(132~274)	Reverse	5'- GTGCTTGCCTCTCACCCTC-3'

Immunofluorescence

Mouse brain sections were washed three times with PBS and antigen retrieval was performed using citrate buffer (pH 7.0); samples were then permeabilized and blocked in PBS containing 0.5% Triton X-100 and 10% normal goat serum at room temperature for 1 h. Sections were incubated with primary antibodies in blocking buffer overnight at 4°C. After washing, secondary antibodies were added to the blocking buffer and incubated for one hour. Samples were then washed, and counterstained with DAPI. Images were acquired using a Nikon confocal microscope.

Primary antibodies used for immunostaining include: Menin (rabbit, 1:1000; Abcam, Ab4452), PV (rabbit, 1:1000; Abcam, Ab11427), GFAP (mouse,1:500; Cell Signaling Technology, #3670), NeuN (mouse, 1:1000; Millipore, #MAB377), Iba1

(Rabbit, 1:300; Abcam, Ab178846), GABA (rabbit, 1:300; Lifespan, #LS-C63353-50), Calbindin (rabbit, 1:1000; Cell Signaling Technology, #2136), 488/594 donkey anti-mouse/rabbit secondary antibodies (1:1000) and Mounting Medium with DAPI was purchased from Invitrogen.

Cycloheximide chase assay

GFP-tagged *MEN1* or *MEN1* mutation constructs were transfected into HEK293T cells treated with cycloheximide (CHX) at 200 mg mL^{-1} at different time points. Proteins were extracted and subjected to western blotting. Turnover of WT-Menin/Menin variants were quantified using Image J.

Plasmids and shRNA

The *shpvalb* vector was constructed by inserting shRNA hairpin sequences into a mU6pro vector. The *shpvalb* sequence is as follows: gcagactccttcgaccacaaa, the primer sequence is as follows: F: ccgggcagactccttcgaccacaaattcaagagatttgtggtcgaaggagtctgc-ttttt; R: aattaaaaagcagactccttcgaccacaaatctcttgaatttgtggtcgaaggagtctgc.

Supplementary Dataset 1

Differentially expressed genes (DEGs) identified from transcriptome sequencing of cortex of 2-month-old G503D mice and controls.

Supplementary Dataset 2

Differentially expressed genes (DEGs) identified from transcriptome sequencing of *Men1* knockout and WT PV neurons.

Supplemental Figures and Figure Legends:

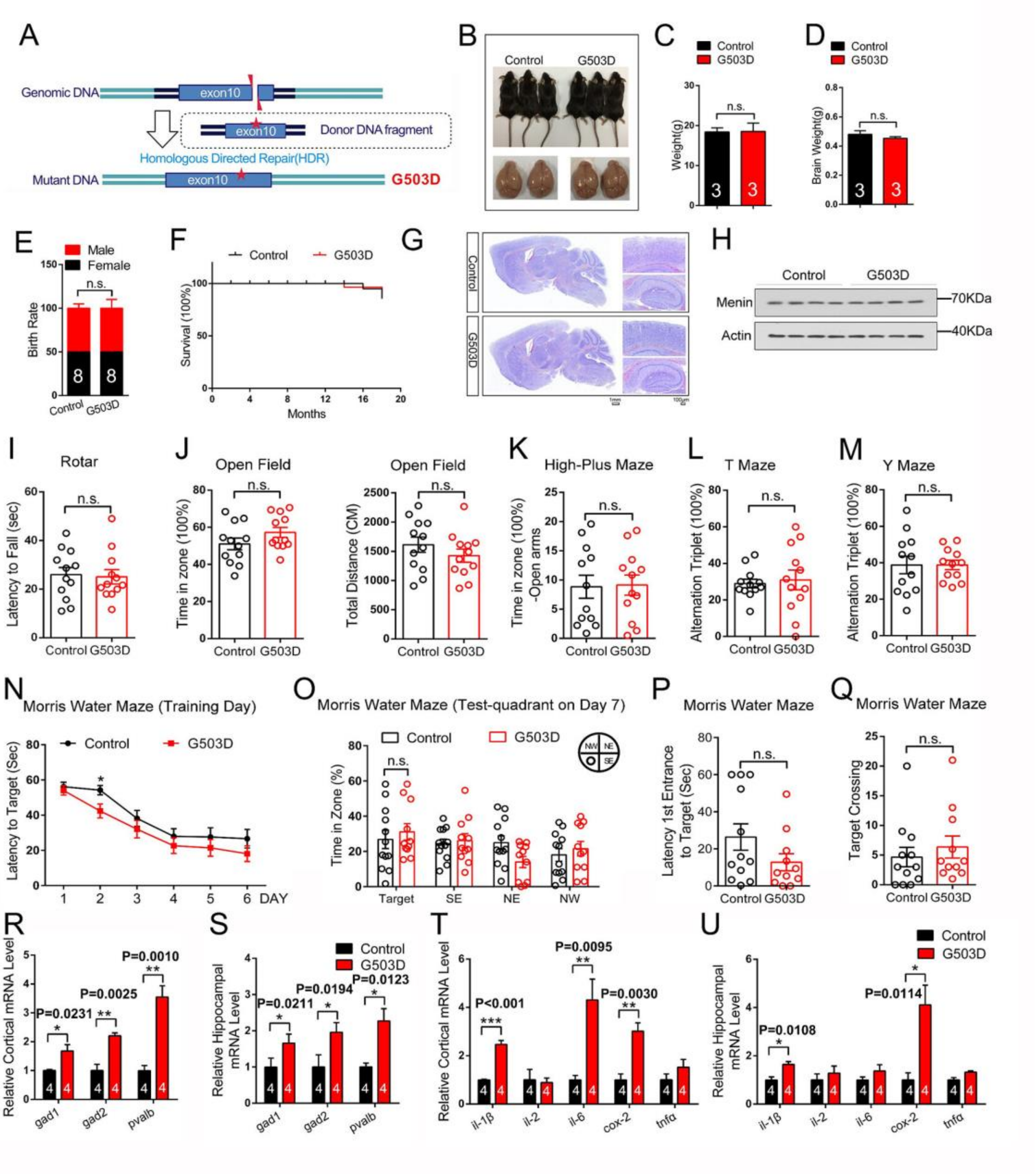

Figure S1.

The generation of Menin-G503D mice, and G503D mice exhibit normally in rotarod, open field, high-plus maze, T/Y maze and Morris water maze tests, related to Figure 1

(A) Generation of G503D mice by CRISPR Cas9. (B-D) Body weight and brain weight of 2-month-old G503D mice and littermate control mice (body weight: control：n=3 mice；G503D: n=3 mice; brain weight: control: n=3 mice；G503D: n=3 mice). (E) Birth rate of male and female in G503D mice and control mice (control: n=8 births；G503D: n=8 births). (F) Survival curve of G503D mice and control mice. Control mice, n = 20; G503D, n = 20. Survival was analyzed for significance using log rank (Mantel-Cox) test. (G) Brain sections from postnatal 2-month G503D mice and control mice were subjected to Nissl staining. Scale bar: 1mm, 100μm, respectively. n =3 mice. (H) Western blotting to test the Menin expression in the cortex of G503D mice and control

mice. n =3 mice. Actin serves as a loading control. (I-M) Behavioral analysis of G503D mice and littermate controls using Rotarod test (I), Open field test (J), Plus maze (K), T maze (L), and Y maze (M). (N-Q) During Morris water maze tests, G503D mice and littermate controls were analyzed for escape latency during a 6 days training period (N). On the next day, mice were analyzed for time spent in the target zone and other quadrants (northeast, southeast, and northwest) (O), time required from entrance to the target platform and number of target crossings (P, Q). (R, S) Quantitative RT-PCR measurements to determine *gad1*, *gad2* and *pvalb* levels in cortex and hippocampus of G503D mice and control mice. n = 4 mice. (T, U) Quantitative RT-PCR measurements to determine inflammatory cytokines expression levels in the cortex and hippocampus from G503D mice and littermate control mice. n = 4 mice.

Mouse number used in behavior tests: Control: n=12 mice, G503D: n=12 mice.

Data represent mean ± SEM, n.s.: not significant, *p<0.05, **p<0.01, ***p<0.001. Kaplan-Meier survival estimate for survival curve. Unpaired t-test for behavioral statistics. Other statistical application between groups were analyzed by one-way ANOVA with Tukey’s post hoc analysis.

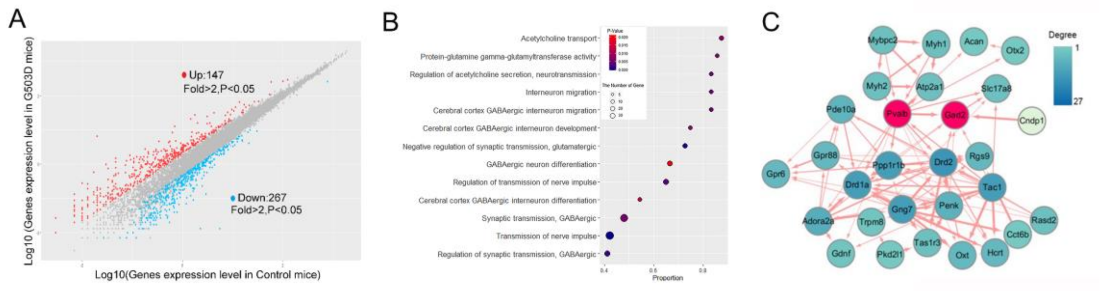

Figure S2.

Transcriptome analysis of cortex from G503D and controls mice, related to Figure 1

(A-C) Differentially expressed genes (DEGs) identified from cortex of 2-month-old G503D mice and controls. We sequenced and analyzed the cortex of G503D mice and littermate controls to get 414 differentially expressed genes (DEGs) (A). Red plots represent upregulated DEGs. Blue plots represent downregulated DEGs. Gray points represent non-DEGs. DEGs were then compared with the nervous system database of mouse genome information (MGI, website: http://www.information.jax.org). By screening the overlapped parts, we carried out to get 111 genes, which were analyzed by GO pathway (B). Interaction network was shown in (C). The detailed RNA-Seq data has been deposited in SPA database, and can be found in PRJNA612763.

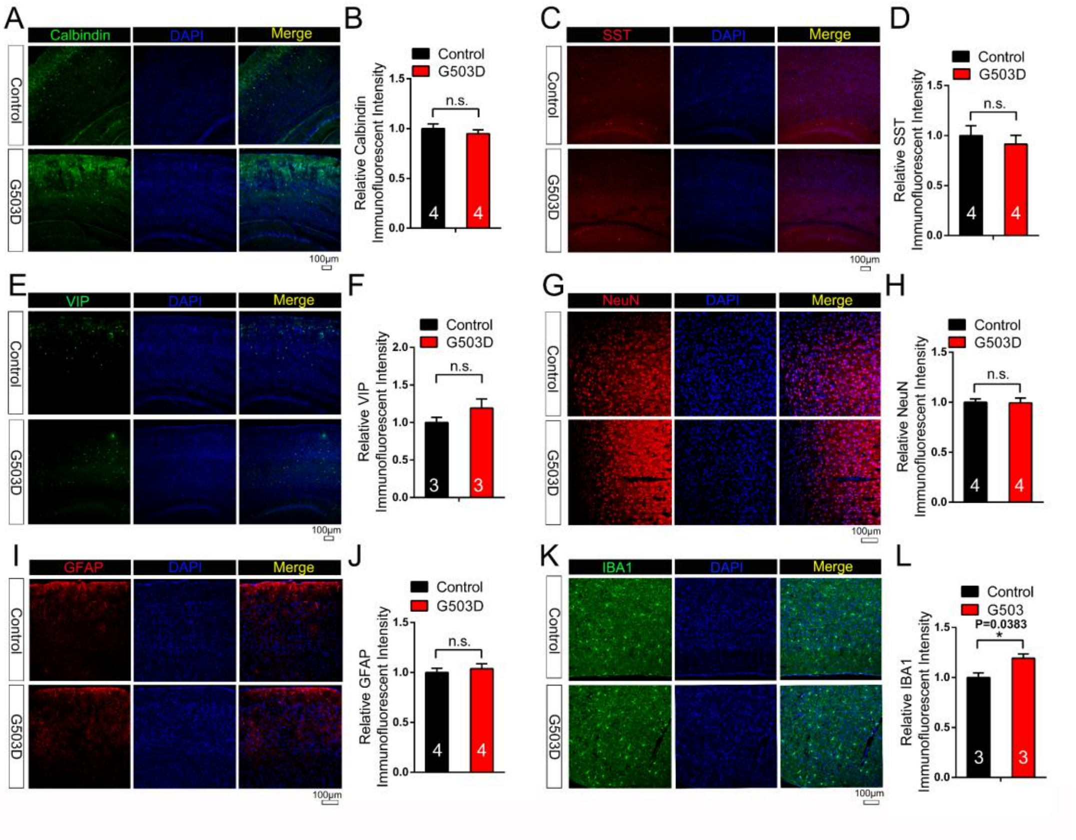

Figure S3.

The immunostaining of calbindin, SST, VIP, NeuN, GFAP and Iba1 in cortex from G503D mice and control mice, related to Figure 1

(A, B) Immunofluorescence of calbindin in cortex from 2-month-old G503D mice and controls. Representative confocal images are shown in panel (A), Scale bar:100μm. Quantitation of fluorescence intensity of calbindin is shown in panel (B), n=4 mice. (C, D) Immunofluorescence of SST in cortex from 2-month-old G503D mice and controls. Representative confocal images are shown in panel (C), Scale bar:100μm. Quantitation of fluorescence intensity of SST is shown in panel (D), n=4 mice. (E, F) Immunofluorescence of VIP in cortex from 2-month-old G503D mice and controls. Representative confocal images are shown in panel (E), Scale bar:100μm. Quantitation of fluorescence intensity of VIP is shown in panel (F), n=3 mice. (G, H) Immunofluorescence of NeuN in cortex from 2-month-old G503D mice and controls. Representative confocal images are shown in panel (G), Scale bar:100μm. Quantitation of fluorescence intensity of NeuN is shown in panel (H), n=4 mice. (I, J) Immunofluorescence of GFAP in cortex from 2-month-old G503D mice and controls. Representative confocal images are shown in panel (I), Scale bar:100μm. Quantitation of fluorescence intensity of GFAP is shown in panel (J), n=4 mice. (K, L) Immunofluorescence of Iba1 in cortex from 2-month-old G503D mice and controls. Representative confocal images are shown in panel (K), Scale bar:100μm. Quantitation of fluorescence intensity of Iba1 is shown in panel (L), n=3 mice.

Data represent mean ± SEM, n.s.: not significant, *p<0.05, **p<0.01, ***p<0.001, one-way ANOVA with Tukey's post hoc analysis.

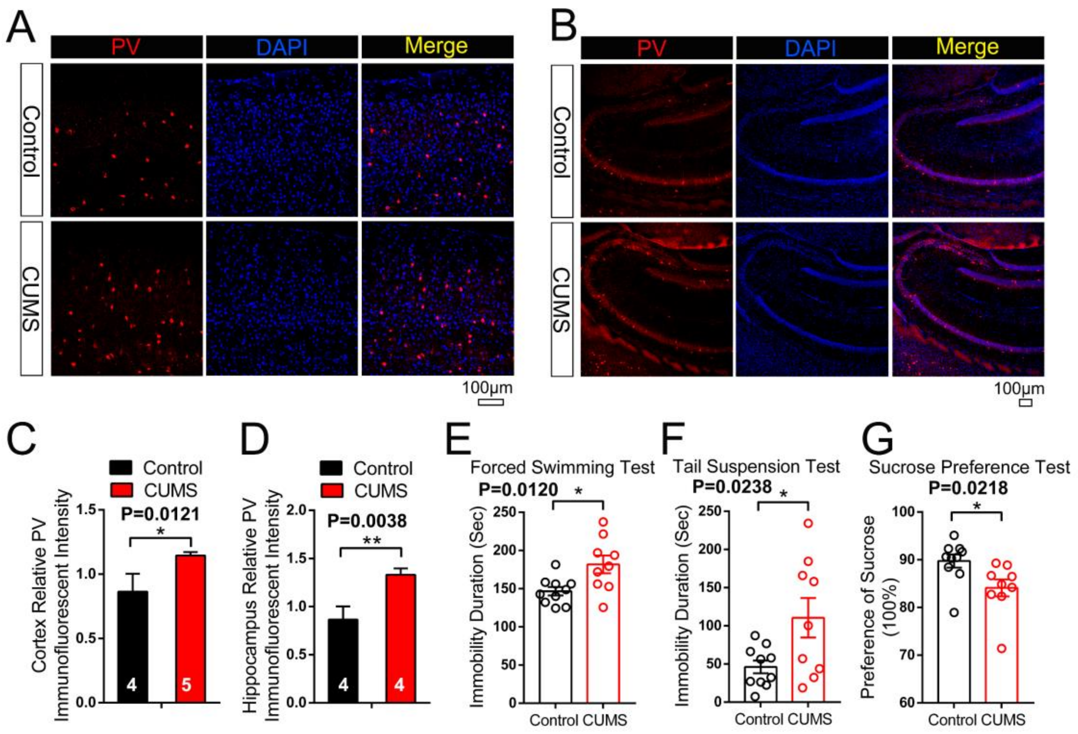

Figure S4.

The immunostaining of PV in cortex from CUMS treated mice and controls, and the depression related behaviors tests of CUMS and control mice, related to Figure 1

(A-D) Immunofluorescence of PV levels in cortex and hippocampus from 2-month-old CUMS mice and controls. Representative confocal images are shown in panel (A, B), Scale bar:100μm. Quantitation of fluorescence intensity of PV is shown in (C, D), n=4-5 mice. (E) Forced Swimming Tests (FST) of CUMS mice and littermate controls. (F) Tail Suspension Tests (TST) of CUMS mice and littermate controls. (G) Sucrose Preference Tests (SPT) of CUMS mice and littermate controls.

Mouse number used in behavior tests: Control: n=10 mice, CUMS: n=9 mice.

Data represent mean ± SEM, n.s.: not significant, *p<0.05, **p<0.01, ***p<0.001. Unpaired t-test for behavioral statistics. Other statistical application between groups were analyzed by one-way ANOVA with Tukey's post hoc analysis.

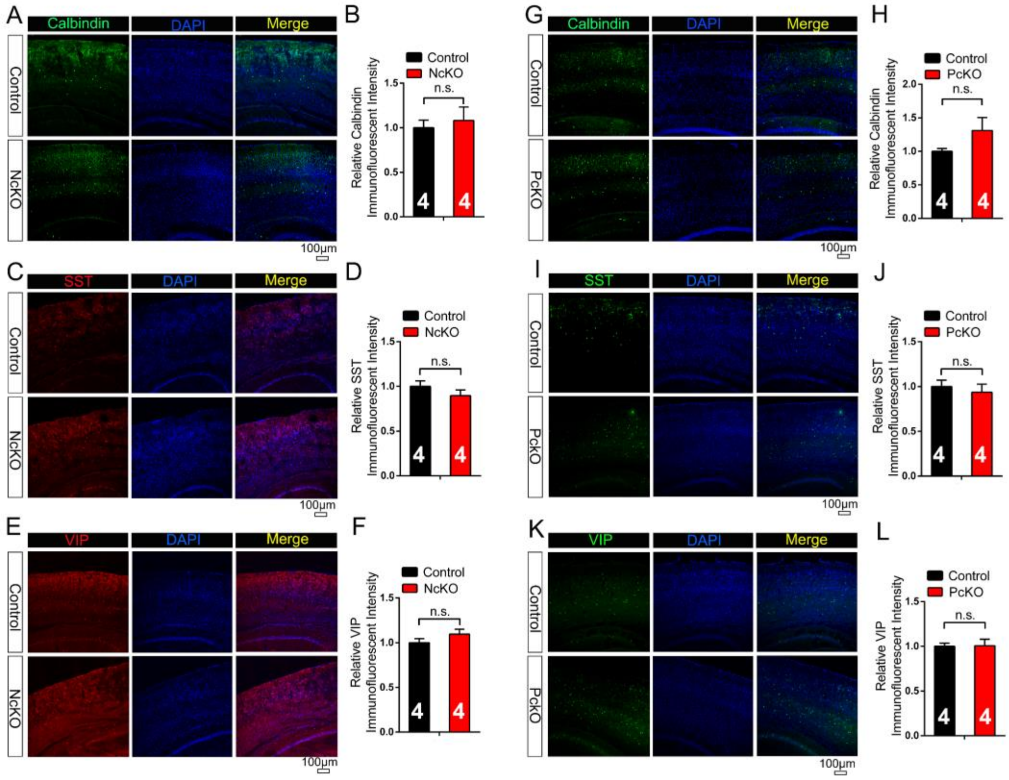

Figure S5.

The expression of calbindin, SST and VIP in *NcKO* or *PcKO* and their control mouse cortex, related to Figure 2

(A-F) Immunofluorescence of calbindin (A, B), SST (C, D), and VIP (E, F) in cortex from 2-month-old *NcKO* mice and controls. Representative confocal images are shown in panel (A, C, E), respectively. Scale bar:100µm. Quantitation of fluorescence intensity of calbindin, SST, and VIP are shown in panel (B, D, F), respectively. n=4 mice. (G-L) Immunofluorescence of calbindin (G, H), SST (I, J), and VIP (K, L) in cortex from 2-month-old *PcKO* mice and controls. Representative confocal images are shown in panel (G, I, K), respectively. Scale bar:100µm. Quantitation of fluorescence intensity of calbindin, SST, and VIP are shown in panel (H, J, L), respectively. n=4 mice.

Data represent mean ± SEM, n.s.: not significant, one-way ANOVA with Tukey's post hoc analysis.

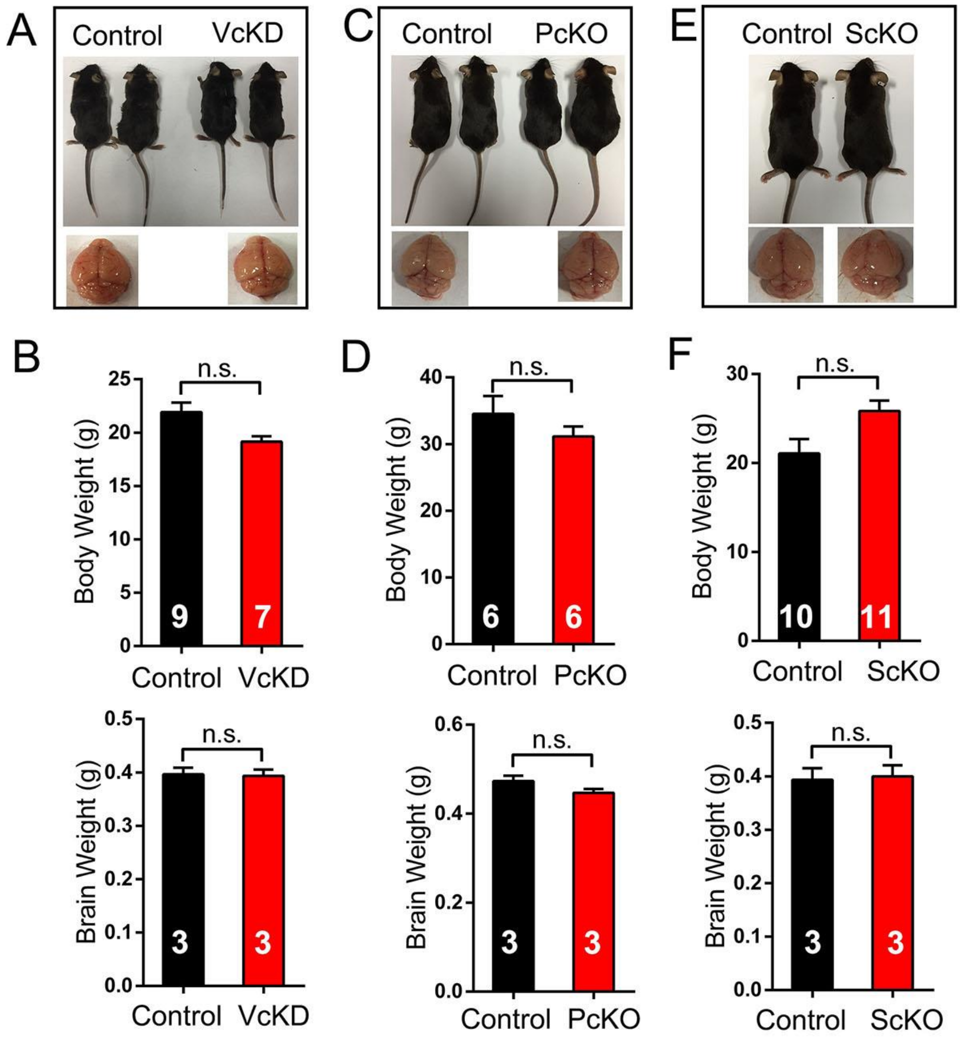

Figure S6.

The body and brain weight of VcKD, PcKO and ScKO mice, related to Figure 2

(A, B) Body weight (n=7-9 mice) and brain weight (n=3 mice) of 2-month-old *VcKD* mice and littermate control mice. (C, D) Body weight (n=6 mice) and brain weight (n=3 mice) of 2-month-old *PcKO* mice and littermate control mice. (E, F) Body weight (n=10-11 mice) and brain weight (n=3 mice) of 2-month-old *ScKO* mice and littermate control mice.

Data represent mean ± SEM, n.s.: not significant, one-way ANOVA with Tukey's post hoc analysis.

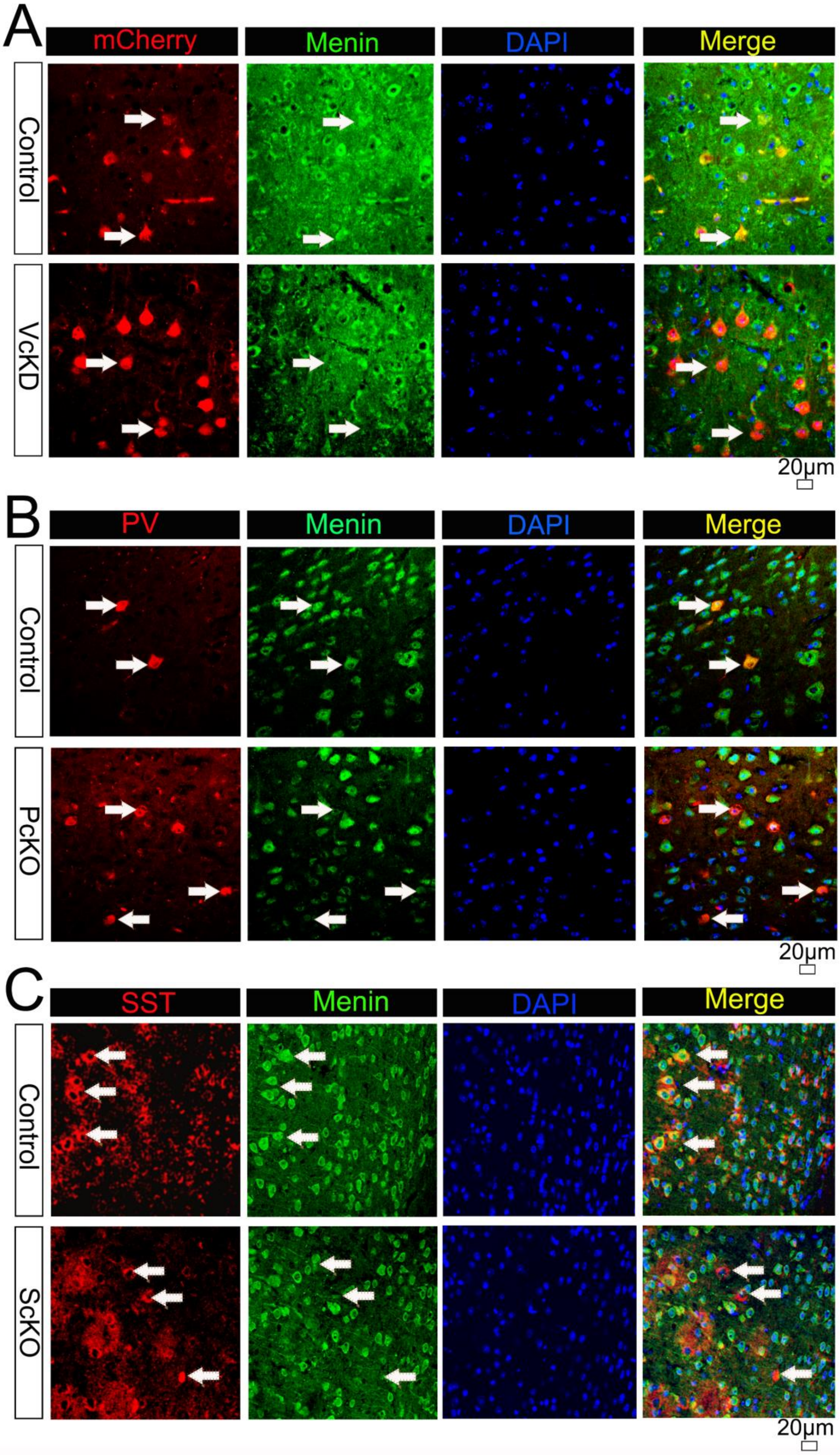

A
mCherry
Menin
DAPI
Merge
Control
VcKD
20μm
B
PV
Menin
DAPI
Merge
Control
PcKO
20μm
C
SST
Menin
DAPI
Merge
Control
ScKO
20μm

Figure S7.

Validation of Menin knockdown efficiency in *VcKD*, *PcKO* and *ScKO* mice, related to Figure 2

(A) Immunofluorescence staining to detect Menin (green) and mCherry (red) in *VcKD* and control mice cortex; Menin expression is dramatically reduced in interneurons in *VcKD* mouse cortex, Scale bar: 20μm. n=3 mice. (B) Double immunofluorescence staining to detect Menin (green) and PV (red) in *PcKO* and control mouse cortex; Menin expression is markedly reduced in PV interneurons in *PcKO* mouse cortex, Scale bar: 20μm. n=3 mice. (C) Double immunofluorescence staining to detect Menin (green) and SST (red) in *ScKO* and control mice cortex. Menin expression is markedly reduced in SST interneurons in *ScKO* mouse cortex. Scale bar:20μm. n=3 mice.

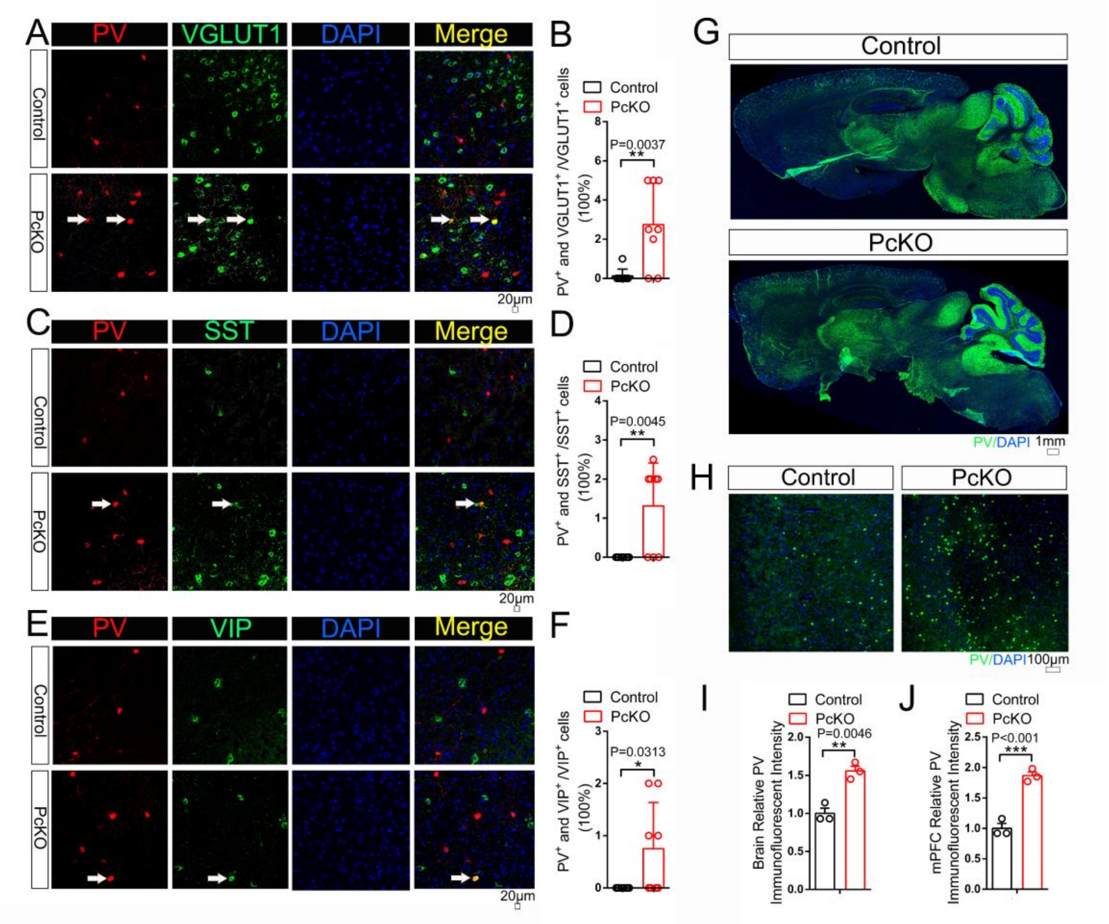

Figure S8.

PV protein is found to be expressed in different type neurons in the cortex of PcKO mice and elevates in brains of PcKO mice, related to Figure 2

(A, B) Immunofluorescence of PV (red) and VGLUT1 (green) in cortex from *PcKO* mice and controls. Quantitation of percentage of PV^+ and $VGLUT1^+$ / $VGLUT1^+$ cells is shown in panel (B). Scale bar: 20μm. n=3 mice. (C, D) Immunofluorescence of PV (red) and SST (green) in cortex from *PcKO* mice and controls. Quantitation of

percentage of PV^+ and SST^+ / SST^+ cells is shown in panel (D). Scale bar: 20μm. n=3 mice. (E, F) Immunofluorescence of PV (red) and VIP (green) in cortex from *PcKO* mice and controls. Quantitation of percentage of PV^+ and VIP^+ / VIP^+ cells is shown in panel (F). Scale bar: 20μm. n=3 mice. (G, I) Immunofluorescence of PV (green) and DAPI (blue) in the brain of *PcKO* mice and controls. Quantitation of PV immunofluorescence intensity is shown in panel (I). Scale bar:1mm. n=3 mice. (H, J) Immunofluorescence of PV (green) and DAPI (blue) in the mPFC of *PcKO* mice and controls. Quantitation of PV immunofluorescence intensity is shown in panel (J). Scale bar:100μm. n=3 mice.

Data represent mean±SEM, n.s.: not significant, one-way ANOVA with Tukey's post hoc analysis.

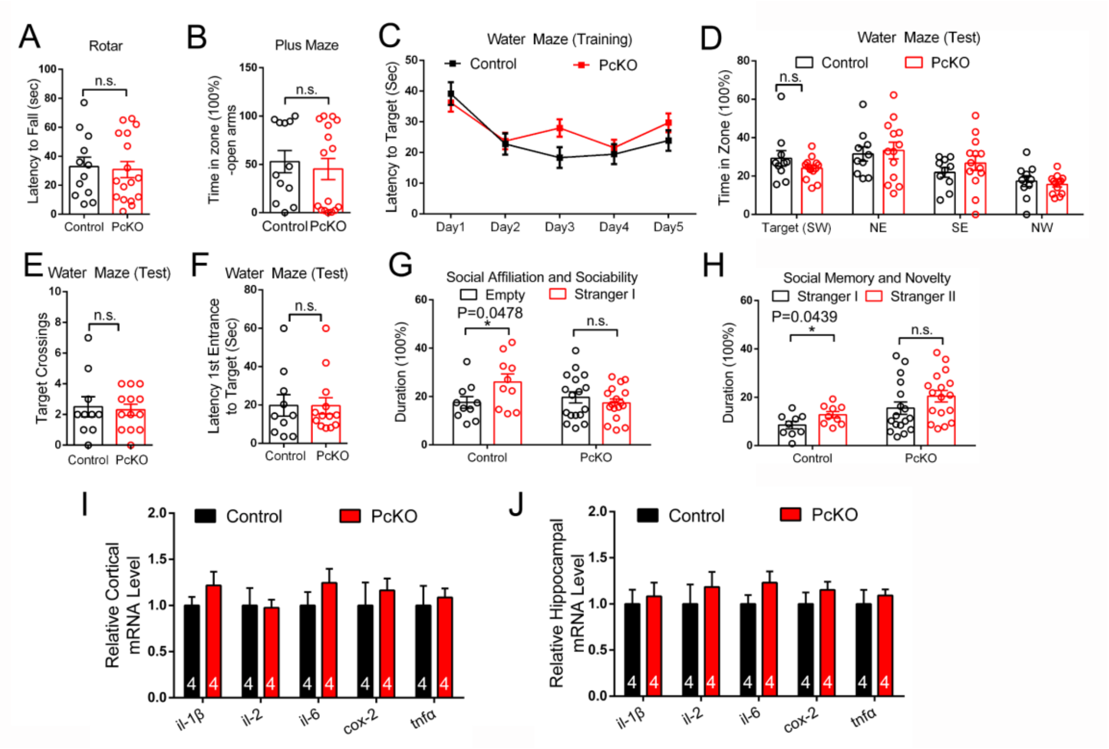

Figure S9.

PcKO mice exhibited no difference in motor ability, anxiety state, cognition and memory abilities compared to controls, related to Figure 2

(A) Rotarod of *PcKO* and littermate controls. (B) Plus maze of *PcKO* and littermate controls. (C-E) During Morris water maze tests, *PcKO* and littermate controls were analyzed for escape latency during a 6 days training period (C). On the next day, mice were analyzed for time spent in the target zone and other quadrants (northeast, southeast, and northwest) (D), time required from entrance to the target platform and number of target crossings (E). (F, G) Social Interaction Tests (SIT) of *PcKO* and littermate controls. Social affiliation and sociability. Mean duration in the chamber with a novel mouse ("Stranger I"-containing chamber) compared to the opposite chamber ("Empty"

chamber). Social memory and novelty. Mean duration spent in the chamber with the mouse from the sociability phase (“Stranger I”), and in the opposite chamber with a new unfamiliar mouse (“Stranger II”). (I, J) Quantitative RT-PCR measurements to determine inflammatory cytokines expression levels in the cortex and hippocampus from *PcKO* mice and littermate control mice. n = 4 mice.

Mouse number used in behavior tests: Control: n=11 mice, *PcKO*: n=16 mice.

Data represent mean ± SEM, n.s.: not significant, *p<0.05, **p<0.01, ***p<0.001. Unpaired t-test for behavioral statistics.

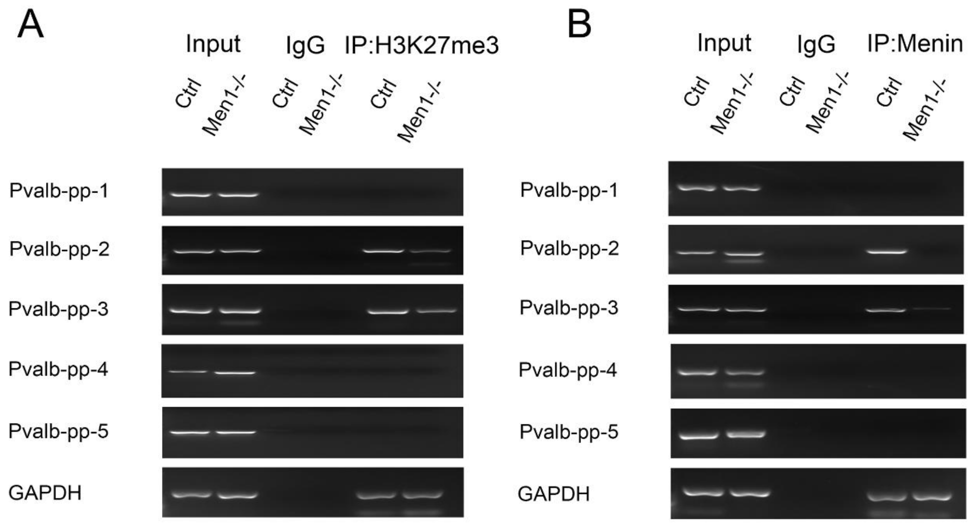

Figure S10.

Representative gel band images of ChIP assays, related to Figure 3

ChIP assays using one of the antibodies against H3K27me3(A) and Menin (B) in wild type primary neurons and *Men1*-null primary neurons (DIV 12). PCR was carried out using primers for each amplicon.

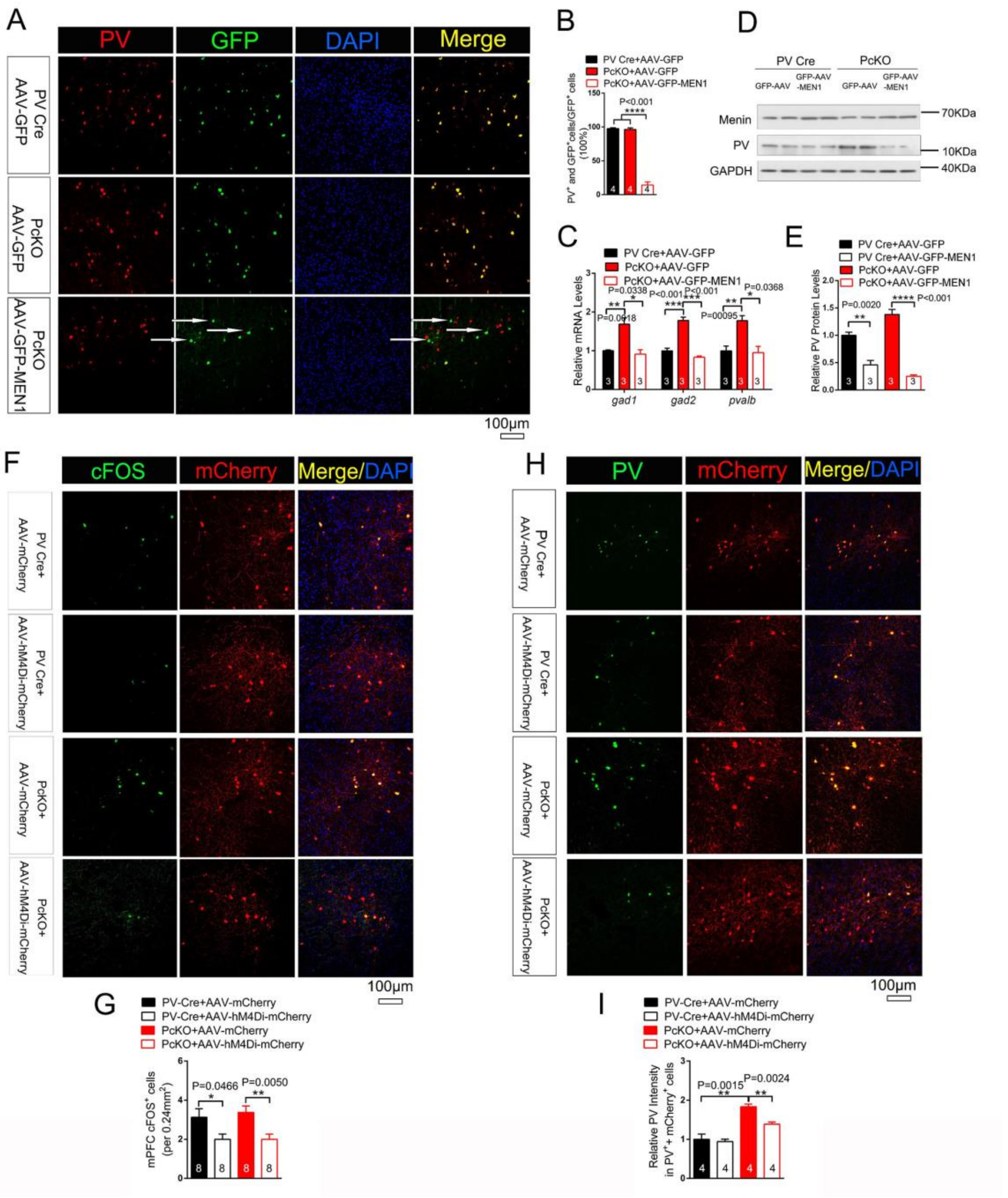

Figure S11.

Validation of the effect of Menin restoration or PV neuronal activity inhibition in PV neurons, related to Figure 4

(A, B) Double immunofluorescence staining to detect GFP (green) and PV (red) in PV Cre+AAV-GFP, PcKO+AAV-GFP and PcKO+AAV-GFP-*MEN1* mice cortex. Representative confocal images are shown in panel (A), Scale bar:100μm. Quantitation of fluorescence intensity of PV in GFP^+ cells is shown in panel (B). n=4 slices from 3 mice. (C) The mRNA levels of *pvalb*, *gad1* and *gad2* were measured by quantitative RT-PCR in the cortex of the above mice, n=3 mice. (D, E) Western blot analysis of protein expression in cortex from PV Cre + AAV-GFP, PcKO+AAV-GFP, PV Cre+AAV-GFP-*MEN1* and PcKO+AAV-GFP-*MEN1* mice. Immunoblots were probed with antibodies against the indicated proteins. Quantification of protein levels is shown in panel (E). n=3 mice. (F, G) Double immunofluorescence staining to detect mCherry (red) and cFOS (green) in PV Cre+AAV-mCherry, *PcKO*+AAV-mCherry, PV Cre+AAV-hM4Di-mCherry and *PcKO*+AAV-hM4Di-mCherry mice cortex. Representative confocal images are shown in panel (F), Scale bar:100μm. Quantitation of $cFOS^+$ cell number is shown in panel (G), n=8 slices from 3 mice. (H, I) Double immunofluorescence staining to detect mCherry (red) and PV (green) in PV Cre+AAV-mCherry, *PcKO*+AAV-mCherry, PV Cre+AAV-hM4Di-mCherry and *PcKO*+AAV-hM4Di-mCherry mice cortex. Representative confocal images are shown in panel (H). Scale bar:100μm. Quantitation of fluorescence intensity of PV on $mCherry^+$ cells is shown in (I), n=4 slices from 3 mice.

Data represent mean ± SEM, n.s.: not significant, *p<0.05, **p<0.01, ***p<0.001, one-way ANOVA with Tukey's post hoc analysis.
